# Lentiviral Vector Bioprocessing

**DOI:** 10.3390/v13020268

**Published:** 2021-02-09

**Authors:** Christopher Perry, Andrea C. M. E. Rayat

**Affiliations:** 1 The Advanced Centre for Biochemical Engineering, Department of Biochemical Engineering, University College London, Gower St, London WC1E 6BT, UK; christopher.perry.14@ucl.ac.uk; 2 Division of Advanced Therapies, National Institute for Biological Standards and Control, South Mimms EN6 3QG, UK

**Keywords:** lentiviral vectors, cell and gene therapy, bioprocessing, manufacturing, lentivirus, pseudotyping

## Abstract

Lentiviral vectors (LVs) are potent tools for the delivery of genes of interest into mammalian cells and are now commonly utilised within the growing field of cell and gene therapy for the treatment of monogenic diseases and adoptive therapies such as chimeric antigen T-cell (CAR-T) therapy. This is a comprehensive review of the individual bioprocess operations employed in LV production. We highlight the role of envelope proteins in vector design as well as their impact on the bioprocessing of lentiviral vectors. An overview of the current state of these operations provides opportunities for bioprocess discovery and improvement with emphasis on the considerations for optimal and scalable processing of LV during development and clinical production. Upstream culture for LV generation is described with comparisons on the different transfection methods and various bioreactors for suspension and adherent producer cell cultivation. The purification of LV is examined, evaluating different sequences of downstream process operations for both small- and large-scale production requirements. For scalable operations, a key focus is the development in chromatographic purification in addition to an in-depth examination of the application of tangential flow filtration. A summary of vector quantification and characterisation assays is also presented. Finally, the assessment of the whole bioprocess for LV production is discussed to benefit from the broader understanding of potential interactions of the different process options. This review is aimed to assist in the achievement of high quality, high concentration lentiviral vectors from robust and scalable processes.



**Contents**
1Introduction · · · · · · · · · · · · · · · · · · · · · · · · · · · · · · · · · · · · · · · · · · · · · · · · · · ·22Bioprocessing of Lentiviral Vectors · · · · · · · · · · · · · · · · · · · · · · · · · · · · · · · ·22.1 Pseudotyped Envelope Proteins · · · · · · · · · · · · · · · · · · · · · · · · · · · · · ·43Upstream Bioprocessing of Lentiviral Vectors · · · · · · · · · · · · · · · · · · · · · · ·8
3.1 Cell Lines for LV Production · · · · · · · · · · · · · · · · · · · · · · · · · · · · · · · · ·8
3.2 Transient, Stable and Induced Production · · · · · · · · · · · · · · · · · · · · · ·8
3.3 Upstream Culture to Produce Lentiviral Vectors · · · · · · · · · · · · · · · ·11
   3.3.1 Adherent Culture · · · · · · · · · · · · · · · · · · · · · · · · · · · · · · · · · · · · ·12
   3.3.2 Suspension Culture · · · · · · · · · · · · · · · · · · · · · · · · · · · · · · · · · · ·13
   3.3.3 Perfusion Culture · · · · · · · · · · · · · · · · · · · · · · · · · · · · · · · · · · · · ·14
   3.3.4 Cell Media and Supplements · · · · · · · · · · · · · · · · · · · · · · · · · · ·144Downstream Processing of Lentiviral Vectors · · · · · · · · · · · · · · · · · · · · · ·15
4.1 Vector Filtration: Initial Clarification · · · · · · · · · · · · · · · · · · · · · · · · ·15
4.2 Vector Filtration: Sterile Filtration · · · · · · · · · · · · · · · · · · · · · · · · · · ·17
4.3 Non-Chromatographic Purification · · · · · · · · · · · · · · · · · · · · · · · · · ·17
4.4 Nucleic Acid Reduction · · · · · · · · · · · · · · · · · · · · · · · · · · · · · · · · · · · ·18
4.5      Chromatographic Purification · · · · · · · · · · · · · · · · · · · · · · · · · · · · ·18
   4.5.1 Anion Exchange Chromatography · · · · · · · · · · · · · · · · · · · · · ·22
   4.5.2 Affinity Chromatography · · · · · · · · · · · · · · · · · · · · · · · · · · · · · ·23
   4.5.3 Size Exclusion Chromatography · · · · · · · · · · · · · · · · · · · · · · · ·23
   4.5.4 Steric Exclusion Chromatography · · · · · · · · · · · · · · · · · · · · · · ·24
4.6 Concentration and Buffer Exchange by Tangential Flow Filtration ·24
4.7 Formulation · · · · · · · · · · · · · · · · · · · · · · · · · · · · · · · · · · · · · · · · · · · · · ·285Vector Characterisation and Quality Control · · · · · · · · · · · · · · · · · · · · · · ·296Whole-Bioprocess Assessment of LV Production · · · · · · · · · · · · · · · · · · · ·317Conclusions · · · · · · · · · · · · · · · · · · · · · · · · · · · · · · · · · · · · · · · · · · · · · · · · · · ·34References · · · · · · · · · · · · · · · · · · · · · · · · · · · · · · · · · · · · · · · · · · · · · · · · · · · · · · · · · · · · ·35


## 1. Introduction

Lentiviral vectors (LV) are commonly used in cell and gene therapies for the transfer and integration of transgenes of interest into recipient cells for therapeutic benefit [1]. As vectors, they are capable of transducing dividing and non-dividing cells such as neurons, haematopoietic stem cells and those of the immune system, notably T-cells, delivering transgenes of up to 11 kilobases (kb) in size. LVs represent a major vector of interest for the treatment of monogenic diseases and adoptive cell therapy trials where gene delivery is required, being present in 57% of ex vivo UK Advanced Therapy Medicinal Products (ATMP) [2]. Over 100 ongoing clinical trials in the US, China, EU and Canada are employing lentiviral vectors both for ex vivo modification of cells or in vivo therapy [3]. Overall, the market for LV production is predicted to grow up to $800 M by 2026 [4] as a result of its popularity in clinical trials and the market approval of recent CAR-T therapies, Kymriah and Yescarta.

With continued interest in lentiviral vectored-therapies, demand for efficient LV bioprocessing is growing. Problems during scale-up and production could delay the adoption of lentiviral vectors for clinical and commercial use. Some bioprocessing challenges encountered today are the inability to produce sufficient titres in the upstream coupled with generally low recoveries during downstream processing, resulting in many companies unable to provide enough capacity to satisfy demand at scale [5]. Despite the current issues with developing suitable quantities of vectors, the applications of viral vectors and their bioprocessing is a valuable enterprise. Considering that only the transgene needs to be changed to pivot to another product, the rise of a universal production process is likely. This can be in the form of a packaging cell line whereby the cell constitutively expresses vector components and an envelope protein of choice, awaiting a suitable transfer cassette for stable or transient expression [6]. For established platforms, a producer cell line may be valuable, whereby the cell constitutively expresses all components relevant to vector generation [7]. Such cell lines lend favourable commercial properties in theory, due to the lack of plasmid DNA and transfection step required. Once the optimised upstream and downstream are designed, the viability of developing a platform for rapid transgene exchange and validation is high. Intensified and continuous processing, such as that seen in recombinant protein production, may be beneficial for cost effective vector production.

## 2. Bioprocessing of Lentiviral Vectors

Lentiviral vectors are unique as a result of their physiological and physico-chemical characteristics. LVs are typically based on HIV-1 and share many of its features, such as its spherical shape at 80–120 nm in diameter [8], capsid core and functional enzymes with an envelope derived from the host cell membrane. The innate complexity and sensitivity of the LV particles impose challenges during processing such as particle thermostability (e.g., half life in the range of 7–8 h at 37 °C [9]), sensitivity to freeze–thaw cycles [10,11] requiring rapid processing, salt [8], pH [9,12], shear [13] and buffer osmolarity [14]. Here, the loss of transduction capability due to non-packaged vector or loss of enzyme function or damaged/absent envelope proteins, is problematic as defective particles can co-purify alongside functional vector [9], reducing process capacity. Furthermore, the producer cell type and their viability during production have been implicated in vector stability [15] and thus cellular health must be maintained for high yields.

The development of lentiviral vectors from the wild type virus has required the evaluation of the viral genome and the selection of viral components to ensure safe and efficient gene transfer. The HIV genome is organised into trans elements that code for functional, structural and accessory proteins, such as Gag-Polypeptide, envelope protein and functional enzymes, whilst non-coding cis elements such as long-terminal repeats (LTR) assist in the transcription and packaging of the viral RNA genome into viral particles and indicates the regions for reverse-transcription and integration into the recipient cell [16,17]. As a result, the initial development of lentiviral vectors has seen the separation of cis- and trans-acting elements of HIV-1 onto separate plasmids with the exception of HIV-1 envelope, which was pseudotyped with an alternative envelope protein such as murine leukaemia virus (MLV) amphotropic envelope protein or VSV-G on a third plasmid [1]. Subsequent generations have incorporated successive improvements to enhance titre and safety with removal of unnecessary viral sequences, such as the accessory proteins *vif, vpr, vpu* and *nef* [18]. In the modern third generation packaging system, *tat* was removed and a constitutive promoter replaced U3 in the 5’ LTR of the transfer plasmid to allow transcription without Tat for improved biosafety [19]. To further maintain safety and maintain vector titres, *rev* was provided on an additional plasmid [20]. Therefore, current practice features 3–4 plasmids, with a self-inactivating transfer plasmid containing a transgene of interest flanked by HIV-1 LTRs with a rev response element. This is co-transfected with a packaging plasmid containing the essential trans element Gag-Pol, a third envelope plasmid containing an envelope protein, and a fourth for HIV-1 *rev* if not included elsewhere.

The requirements of LV processing vary with application, but high titres and purity is of main importance. Most impurities in vector production will be that of residual DNA, transfection reagents, host cell proteins and media components (see Table 1). With cell therapies, where the final product is transduced cells, the purity profiles of the vector are less stringent than that for in vivo therapies as Quality Assurance/Quality Control (QA/QC) requirements for product release lie at the cell stage. For in vivo therapies, the requirements will be pertaining to the vector itself. Thus, for an in vivo therapy process, the critical quality attributes would be weighted towards quality and concentration, whereas ex vivo would be quality and yield [21].

The scale of production depends on the number of expectant patients, the quantity of cells needed to be transduced and which multiplicity of infection (MOI) is suitable, inclusive of repeated doses, potency, disease and whether delivery is in vivo or ex vivo. Ideally, MOI should be kept low to ensure single transgene integrations to limit the risk of cytotoxicity and tumorigenic potential [22,23,24]. Ultimately, there is no apparent fixed titre of vector required, but annual transducing units (TU) from manufacturing processes can range from $10^{10}$ to $10^{12}$ TU depending on patient numbers and dosing [25]. The sensitivity of autologous cell therapies to scalable viral production is indicated in some cost of goods analysis, with up to 26% contribution to cost when viral titre is poor [26]. Likewise, cost per dose is further shown to be dependent on titre at harvest and the production method employed [27].

### 2.1. Pseudotyped Envelope Proteins

LVs are commonly pseudotyped whereby the envelope protein of the vector is exchanged with that of another virus (see Table 2), in effect, enrobing the viral particle with proteins from other viruses [28,29] which provide varying characteristics to the vector, affecting its tropism and intended cell target as well as possibly impacting success in bioprocessing. When transducing a cell, the envelope protein must contact and bind to a surface receptor on the recipient cell. The choice of envelope protein, its frequency on the vector surface and the availability of the receptor are crucial for efficient transduction whilst restricting off-target interactions. In LV bioprocessing, the envelope proteins must be suitably expressed in producer cells and incorporated into the vector. As the proteins belong to the vector’s surface, they interact directly with the bulk media and are therefore affected by physico-chemical conditions applied during processing such as shear forces, salt concentrations and pH. As a result, the selection of envelope protein is important for vector design in the function of the vector and its intended target but also on how the vector is processed in terms of its initial expression during upstream processing, its effect on producer cells and its impact during unit operations in downstream processing.

Many viral envelope proteins can be pseudotyped onto LV particles. However, the most commonly pseudotyped envelope protein, and widely considered the gold standard, is the VSV-G glycoprotein from the vesicular stomatitis virus Indiana. This protein is routinely used in part due to its broad tropism, interacting with low-density lipoprotein receptors (LDL-R) which are ubiquitous in most cell types [68,69]. However, from a vector design perspective VSV-G has the disadvantage of displaying cytotoxicity, resulting in cell instability in LV producer cell lines when highly expressed [13] and limiting its application to transient transfection modalities. Although stable expression of VSV-G has been illustrated [37], it is likely the effect of vector superinfection or reduced expression resulting in poor results. While inducible promoters for VSV-G may avoid cytotoxicity from constitutive expression as described in [70], this may be problematic during processing due to the addition or removal of inducing agents (see Section 3.2). VSV-G LVs are typically applied in ex vivo therapies due to their inactivation by complement in human serum [71]. Additional immune responses against VSV-G are apparent in vivo [72] which can inhibit subsequent LV administration by adaptive immune responses [73]. Moreover, despite the ubiquity of LDL-R, clinically valuable resting lymphocytes for CAR-T therapies have particularly low expression of the receptor, and thus transduction is inefficient unless activated prior [74]. However, due to the high functional titres VSV-G provides, its application is widespread and is a common envelope protein used.

Envelope proteins are impacted by physico-chemical conditions that need to be considered in downstream processing. LVs with VSV-G envelopes have been observed to inactivate when pH diverges from pH 7 [9], and when treated with 1 M NaCl, where 50% of functional titre is lost within 1 h [8]. The implications for vector processing are particularly pronounced in ultracentrifugation, whereby LVs with the envelope protein VSV-G outperform those with influenza envelope proteins [75] or HIV-1 envelopes over VSV-G [13,76] and appear to be resistant to shear [13,76,77]. Robustness of envelope proteins during processing will need to be considered and to account for discrepancies across vector envelope stability.

Other envelope proteins have been investigated and applied for LV pseudotyping. The glycoprotein Cocal-G deriving from the same vesiculovirus family as VSV shares 71.5% of amino acids sequences to VSV-G, and therefore has similar characteristics [36]. Transduction by Cocal-G enveloped LVs has been inhibited by soluble LDL-R, similar to VSV-G, and is therefore likely to infect via the same LDL-R or similar receptors [36,68]. Unlike VSV-G, Cocal-G is not inactivated by human serum and can be expressed constitutively allowing the potential for stable expression in vector producing cell lines [36,78], although this does lead to superinfection and cell instability [37]. Additional vesiculovirus family glycoproteins have been examined for stable producer cell line generation, such as those from PIRY, Chandipura and VSV-New Jersey, which displayed titres of $10^{5}$ to $10^{6}$ TU/mL and robustness during concentration and freeze–thaw [37,79]. In addition, the viral envelope protein RD114 has been applied in vector production. Deriving from a feline endogenous retrovirus, the RD114 envelope shows less cytotoxicity, is not inactivated by complement and allows for usage in stable producer cell lines [30,32,80,81]. These envelope proteins are capable of transducing CD34+ cells and show robustness during ultracentrifugation with 50–70% yield with 100–200-fold concentration [30,82,83]. RD114 has further been modified, with the insertion at the R peptide cleavage site of the HIV matrix/capsid cleavage sequence, thus giving rise to the RDPro envelope protein, which has shown a log order higher titre in transient production compared to RD114 [34,84]. These RD114 derived envelope proteins further prevent the action of superinfection, whereby a produced vector is blocked from transducing the cell line it originated from [37] and has found application in the transduction of lymphocytes for CAR-T therapy [85,86] and CD34+ progenitor cells [35,87]. Other envelopes, such as Sendai, require trypsin treatment to activate the envelope by protein cleavage [88,89], although the supplementation of an additional unit operation will need to be considered. Moreover, Sendai activated envelopes perform worse during ultracentrifugation, dropping to 20% recovery compared to 50% of non-cleaved envelope [11].

With pseudotyping of viral vector, theoretically, any viral component that is expressed on a cell surface can be used. However, such methods rely on viral proteins, which can raise concerns on safety and development costs particularly on screening. A method that has been applied is “plug and play” envelope proteins, whereby LVs are targeted to specific cell types by covalently bonding cell targeting proteins onto their surface via a disulphide bond [90] or by biotinylated targeting ligands [91]. Such work may improve the possible targeting mechanism for vectors, although this may require additional unit operations during processing to bond targeting components fully.

## 3. Upstream Bioprocessing of Lentiviral Vectors

The focus of upstream processing is to mass produce LV in bulk while allowing efficient downstream purification. There are several important factors to manage which are dependent on the production mode, either by transient transfection of cells by plasmids or using a stable or inducible producer cell line. A fundamental factor is the scalable expansion of cells, their viability and achieving suitable cell densities for optimal LV generation. Therefore, the type of cell line, either adherent or suspension, the bioreactor for expansion, the type of cell media with suitable supplementation and the method of transient transfection if applicable are important factors to consider.

### 3.1. Cell Lines for LV Production

A suitable cell line is required to produce LV particles at a high titre. Commonly, the cell line of choice is the human embryonic kidney cell 293 (HEK-293) [92], specifically the derived 293T line [93]. HEK-293Ts have the SV40 T-antigen, which is implicated in inhibiting p53 [94] and preventing the activation of the intracellular innate immune response, and it has been shown to boost titres of LVs [95]. This latter cell line has shown several advantages over its precursors, notably a shorter doubling time, higher transfection efficiencies and higher vector titre [96,97] as well as adaptation to suspension culture [98]. The ability to grow dense cultures of cells is highly beneficial for high vector titres, as each cell is theoretically a production unit for LV [99]. This however requires a degree of optimisation in terms of the overall process, as high cell density is tied with lower cell viabilities, which will contribute to greater masses of contaminants to be removed during processing. This caveat may negate any benefits to titre that greater cell numbers provide [100].

### 3.2. Transient, Stable and Induced Production

The dominant LV production mode is the transient transfection of cells with a variety of plasmids for LV expression and packaging. Initially developed to minimise the risk of replication competent vectors, this method has seen generational development to improve yield and safety. Plasmids are co-transfected with chemical transfection agents, and typically give titres in the range of $10^{6}$ to $10^{7}$ TU/mL non-purified [97], although bigger transgenes give lower titres [101].

Despite this, contamination of the feed with residual plasmid DNA and transfection reagent is of concern and removal of these are necessary [102,103,104]. Media exchange post-transfection is normally mandatory due to cytotoxicity issues by transfection agents, requiring additional equipment for pumps and tanks to mix plasmid DNA, transfection reagents and media, in addition to increasing overall media consumption [105]. Moreover, many factors can affect the efficiency of transfection, including plasmid concentration, reagent:DNA ratio, cell density, incubation time, mixing regime, temperature and pH [106,107], some of which are challenging on scale up. As a result, significant batch-to-batch variability with vector titre is apparent [99]. The cost of clinical grade plasmids and transfection agents adds significant expense to a production run, with at least 1µg plasmid DNA per million cells being used typically [99].

There are a multitude of transfection reagents available for LV production, the choice of which will drastically affect the processing step and cost of goods for a production run. Calcium phosphate (CaPi) is an economical transfection reagent commonly used at lab or small scale that entails the formation of fine precipitates of calcium-phosphate and DNA upon mixing calcium chloride and DNA with HEPES buffered saline. The precipitates settle and are endocytosed by cells [1,108]. However, this method is difficult to scale due to the mixing of two buffers to produce consistent precipitates; with variables such as component concentrations, temperature and aggregation time requiring optimising [109]. The process is further complicated due to slight variations in pH which negatively affect transfection efficiency [110]. In addition, CaPi transfection may cause cytotoxicity in cells without the protective action of serum or albumin in the cell culture [111,112].

Cationic lipids such as lipofectamine have also been used [113]. They form liposomes that entrap plasmid DNA by complexing with its negative charge. The liposomes fuse with the cell membrane releasing its contents for expression. Although capable of transfecting many cell types, lipofectamine is cytotoxic and can lower viability [114] and thus media exchanges are required after application [115], and are therefore typically used only at development scale.

Polyethylenimine (PEI) is more suited to scalable transfection and offers an efficient and cost-effective choice by forming polyplexes with DNA that are endocytosed by cells [106,116]. The formation and degradation of polyplexes is a function of time, and therefore there is an optimal window for use. Despite this, PEI is considered simpler than CaPi and does not require as strict controls for optimal transfection whilst also using less plasmid DNA [117]. Recombinant baculoviruses have also been used as transfection reagents to transfer viral components [118,119], although this may incur greater costs to separate baculovirus from lentiviral vector in downstream processing (DSP). Regardless of method, vector production typically peaks within 48hr post-transfection and declines from 72 h onwards providing a limited time window for harvesting. Ideally, the vector population would be of similar quality, although, due to variable transfection efficiencies on a per cell scale and their impact on viability, the vector population is typically heterologous [15].

An alternative to transient transfection can be found in stable producer or packaging cell lines (see Table 3). In these, the viral components are constitutively expressed within the cell and do not require additional plasmids and transfection reagents, except for packaging which only requires the transfer plasmid [6]. This simplifies the upstream culture and avoids the additional cost and contaminant load from residual plasmid. Moreover, stable cell line producers may generate a greater quality of particles, due to the clonal source of production allowing near homologous particles to be produced. Furthermore, such harvests tend to be cleaner [120], likely due to less envelope-based vesicles present, in addition to absent plasmid and transfection reagent load. Such stable cell lines have the potential to offer a cost effective, scalable and reproducible vector run with less batch to batch variability as each cell would maintain consistent productivity.

High performing stable producers have been created via genome tagging, screening for active loci and replacing it with lentiviral components [127,128]. Cell lines such as WinPac [7] and LentiPro26 [121], which provide titres in the $10^{6}$ TU/mL/day range, have been developed with the former utilising a retroviral tagging and recombinase mediated cassette exchange for high Gag-Pol expression and the latter applying a less active mutated viral protease to maintain cell viability and improve titres. These stable producer cell lines can be arduous to develop, requiring the isolation and evaluation of individual clones for component expression that require culturing in selection antibiotics to maintain titres. Efforts have been developed to streamline this process such as the use of bacterial artificial chromosomes which incorporate all vector components on a singular construct [129,130,131] or developing high-throughput screening methods such as the co-culturing of singular isolated producer cells encoding vectors with partial GFP fragments and cells expressing the complementary GFP fragment and monitoring for GFP reconstitution to identify high producers [132]. However, critically, stable producer cells offer titres lower than those of transient led methods, and therefore their adoption is problematic. This is particularly due to the cytotoxic effects that viral protein expression can have on cells, such as the protease, which has been linked to cleaving pro-apoptotic proteins [133], and the limits on choice of envelope protein. As an example, VSV-G, a highly effective envelope protein, is difficult to express constitutively. Potentially cytotoxic transgenes are also problematic for long term expression in stable producers. This has been remedied via a bacterial tryptophan RNA-binding attenuation protein which blocks transgene expression by binding upstream of the ribosome initiation site [134], limiting its expression within the producer cell line.

Typically, the greatest challenge for producer or packaging cells is managing the cytotoxic or cytostatic effects while maintaining high titre. Whilst this can be mitigated with inducible systems and have been accomplished with LV [126,135,136], these methods are currently not wholly practical. Tet-on induction will require the addition of tetracycline which must be removed at a later point, whereas tet-off induction may require extended culture time for expression to peak as well as requiring a complete media exchange, which may be uneconomical. In all inducible cases, the risk of “leaky” expression is also apparent with non-controlled production of LV being possible.

Nonetheless, with further development, the application of stable producers would be greatly beneficial in the production of LVs. Such improvements can be in the form of high throughput automated clonal isolation and evaluation in addition to improved cassette design, envelope choices and cell line development. Possible strategies to optimise LV production is to up-regulate anti-apoptotic genes, downregulate intracellular sensing and optimise the protein and lipid generation pathway for efficient vector production.

### 3.3. Upstream Culture to Produce Lentiviral Vectors

To produce high titres of LVs, high quantities of cells are required. There are various solutions to expand the number of producing cells (see Table 4) for LVs, pursuant of adherent or suspension cells at varying scales. During development, small batch sizes are desired for flexibility of production and are typically dominated by adherent cultures primarily due to HEK-293T being innately adherent, the high cell densities offered and ease of access. This is usually fulfilled by culture flasks, dishes and bottles. With scale up, where larger and more consistent batch sizes are desired, production transitions to bioreactors in the form of stirred tanks, rocking waves and fixed bed bioreactors with multi-layer flasks straddling the intermediate scales. In terms of efficient cost of goods production, a recent study indicates single-use stirred tanks as most cost-efficient where suspension culture is available, otherwise fixed beds offer greater savings than adherent flask culture [27]. Many upstream culture vessels have also transitioned onto single use disposables. These not only assist in reducing change-over times, but also aid in validation by reducing the cleaning and sterilisation stages for equipment.

#### 3.3.1. Adherent Culture

At development stages, adherent cultures are ideal, however they become limiting with scale. Here, either scale out with multiple flasks or scale up vertically into multilayer flasks is employed. These flasks offer a range of surface areas available for cell attachment and growth with the 1–40 layer CellSTACKS (Corning, 636–25,440cm${}^{2}$) and the 1–40 layer cell factories (Nunc, 632–25,280 cm${}^{2}$) commonly used [97]. In addition, the HYPERflasks (1720 cm${}^{2}$) and HYPERStacks (6000–18,000 cm${}^{2}$) (both Corning) offer gas-permeable plastic for the mass transfer of O${}_{2}$ and CO${}_{2}$, and therefore do not require as large of a headspace for vector production [138]. The scale out or scale up of these systems become increasingly bulky and cumbersome to handle at high surface areas, requiring greater incubator space and transport considerations, often needing additional equipment to assist. With scale-out, there is increased manual handling and risk of contamination from open manipulations, particularly problematic as batches are often pooled for downstream processing [96]. In addition, transfection stages are multiplied, increasing the chance of variable vector production, and adding complexity to technician workload. Adherent culture in flasks and roller bottles do not allow for culture management and are batch-mode in nature, with no in-line ability to monitor and control for dissolved oxygen, pH, waste products and nutrient replenishment.

An alternative culture method for adherent cells are microcarriers, which have been applied to HEK-293 and HEK-293T for retroviral vectors [150] and LV [151]. Such microcarriers can be porous or solid, allowing cell growth within or on the surface. However, microcarriers have been linked to clumping and cell detachment [151]. Furthermore, microenvironments can occur within the centre of the microcarriers, whereby cells on the outer surface limit the mass transfer in of nutrients and oxygen, while limiting the release of toxic metabolites, CO${}_{2}$ and viral particles [152], resulting in low LV titres [150].

For scaling purposes, LV production from adherent cells is often transitioned into fixed bed bioreactors. As an alternative to flask-based or bottle culture, cells are expanded on a 3D matrix composed of highly porous microfibre carriers that offer substantial surface areas at economical volumes. Such reactors have been applied to retroviral and adenoviral vectors [153,154]. Examples of commercialised fixed bed bioreactors are Pall’s iCELLis or Univercells’ Scale-X bioreactors offering surface areas of 0.53–500 and 2.4–600 m${}^{2}$, respectively. Such bioreactors allow for in-line monitoring and control of culture parameters for vector production. In-line monitoring offers greater insight from the bioprocessing environment and raises applications in in silico modelling off-line [155] for future optimisation. Furthermore, the Scale-X process can be intensified as part of Univercells’ Nevoline that offers in-line concentration by Tangential Flow Filtration (TFF) and modular downstream processing options such as clarification and chromatography. The iCELLis has been used to produce transient LV in a perfusion system at fixed perfusion rates or targeting specific glucose targets, ultimately achieving titres above $10^{10}$ TU in a 4 m${}^{2}$ packed bed in perfusion mode (total volume 5.5 L) [144]. Although this work demonstrated that optimisation is still required, as fixed bed reactors are affected by poor cell distribution and may not optimally expand nor allow efficient transfection particularly at greater compaction, ultimately being less productive per square cm than adherent flasks [144]. Similar titres were produced in Scale-X bioreactors where cell distribution was more homogenous [149]. A side-by-side comparison of LVs from iCellis Nano with LVs manufactured under cGMP using 10-layer cell factories has also demonstrated similar transduction efficiencies [156]. Nonetheless, the singular run of an iCellis Nano in this study was equivalent to 30 triple flasks, demonstrating the impact of scale up options within the upstream.

#### 3.3.2. Suspension Culture

Suspension cultures are commonly applied in the production of various recombinant proteins due to their ease of scaling, control over culture parameters and broad industrial familiarity. For LV production, HEK-293T cultures can be adapted to suspension cultures and used in conventional stirred tank bioreactors and rocking bags [99,140]. Suspension cultures provide a solution for scale up, minimising manual handling, allowing perfusion culture, automation, in-line monitoring and control in addition to simplified application of transfection reagents. However, cells must be adapted to suspension culture for use in these bioreactors. This may be difficult to accomplish without loss of productivity, with titres being in the $10^{6}$ TU/mL range and below [157]. Only recently have titres been reported to achieve $10^{8}$ TU/mL via gradual adaptation in suspension media [98]. In general, the cell density is much lower than in adherent, thus vector will be naturally diluted in such methods. Furthermore, additional consideration for initial clarification is required to remove suspended cells.

Stirred tank bioreactors provide the simplest scaling methodology applied to LV production. These units can incorporate development scale productions, such as in Ambr bioreactors [146] for HEK-293T growth optimisation, to larger scale with 10 L Biostat [147], with the latter bioreactor reported to scale up to 10,000 L. Additionally, the Pall Allegro reports titres of $1.1\times 10^{10}$ TU/L [158]. Scale up of stirred tank bioreactors follow typical scaling parameters, such as following kLa volumetric mass transfer coefficients, power volume (P/V) ratios and tip speeds. As mammalian cells typically require less kLa than bacterial cells [159,160], the scaling parameter is favoured towards P/V and tip speed to prevent shear damage to producer cells. Although in terms of scale up, limitations are evident in transient transfection production strategies whereby media replacements may be required which is more difficult with suspension cells [105].

Rocking bioreactors offer a method for the expansion of producer cells in single use inflated disposable bags wherein the cells and media are rocked gently on a platform to promote mixing at scales of 10–200 L. The method offers high mass transfer of gasses whilst giving a low shear environment for cells due to the absence of impellers. Such bioreactors have been used to produce vectors from stable producer cell lines at clinical scale [140], although this run utilised Fibra-Cel disks as growth matrix in this instance, although suspension cells are noted in patent documentation [161].

#### 3.3.3. Perfusion Culture

The option of perfusion culture can remove cell waste products as they develop and maintain nutrient levels for producer cells. The vector residence time within the culture vessel is also reduced, meaning less non-functional vectors are present due to time-sensitive losses, improving yield. Such perfusion methods have allowed up to 15× improvement over batch runs [162] with up to $10^{11}$ cumulative TU per litre [163] and better successes in fixed bed reactors [144]. Furthermore, due to the temperature sensitivity of vectors, perfusion can allow vectors to be absconded to a cooler environment away from the destabilising warmer culture temperatures for cells. Although perfusion cultures require high media consumption depending on set perfusion rate which may increase cost, some monoclonal antibodies (mAb) processes indicate potential cost savings compared to fed-batch methods [164]. A perfusion culture can hypothetically be integrated to lead directly into any downstream process, such as chromatography, for continuous purification and intensified processes.

#### 3.3.4. Cell Media and Supplements

The choice of media directly correlates to the expansion and viability of the producer cells and success during downstream processing as the media is responsible for the vector’s stability and forms the initial vector containing feed to process.

There is a drive to remove serum from the production of LV [98]. Many LV production strategies use serum to assist in optimal cell growth and for the stability of the vector itself, which seems to benefit from high protein solutions [165] likely due to albumin and lipid related stabilisation [166]. Supplementation with albumin shows stabilisation of the vector in DSP processing [167]. However, most serum used are bovine derived and thus bovine related disease transmission and the threat of global shortage are of concern. Whilst recombinant human albumin may possibly act as a substitute, this would add to the cost of production. Moreover, the high protein load adds stress to the downstream process which must remove most of the serum contaminants. Such pressures have led to the use of serum-free media for cell culture, and it is now well established in LV production with resulting titres comparable to serum containing media [98,126,168,169]; HyCell TransFX, FreeStyle 293 and SFM4TransFx-293 are just a few examples of commercially available media in use [170]. Such serum-free expression of vector typically involves the sequential reduction of serum in culture until cells are adapted to low or zero serum concentrations. Otherwise, a common upstream tactic is to expand the cells in serum containing media and exchanging to a serum-free alternative prior to vector harvest.

Supplementation of the cell culture has been connected to improvements of LV titre. Sodium butyrate addition is associated with improved recombinant protein production, via the inhibition of histone deacetylase, leading to improved transcription factor function resulting in increased RNA copies from viral LTRs and improved LV titres [171,172]. Additional supplementation with cholesterol and lipids has been linked to improved titre, likely due to stabilisation of lipid rafts on cell surfaces where virions bud [173,174,175]. However, a study that utilised Sendai F/HN envelope proteins and different media types has found no significant improvement to yield with additional supplementation, but it is still suggested that smaller cumulative effects may be of benefit [176]. There has been some improvements to yield due to the presence of caffeine in cell media post-transfection [177], although such results have not been replicated. Furthermore, it has been reported that decreasing the pH to 6 prior to harvesting leads to a tripling in titre [178]. In this experiment, VSV-G envelope protein was used, and at pH 6 its infectivity is likely reduced, thus potentially reducing the impact of superinfection. It has also been shown that high cell media osmolality is associated with improved retroviral stability via lower cholesterol to phospholipid ratios in the viral membrane, leading to improved stability [14,179].

## 4. Downstream Processing of Lentiviral Vectors

The downstream bioprocessing of LV material concerns itself with maximising vector recovery and minimising components which may negatively impact the efficacy or safety of the product whilst remaining economically viable. Whilst the ultimate product profile is dependent on whether vectors are applied in vivo or ex vivo [21], and the final amount of transducing units is dependent on verified treatment doses, in general, bioprocess operations must reduce the amount of impurities while maintaining viral efficacy to ensure end-user safety. These impurities can be residual DNA (from host cells and plasmid), host cell proteins, serum, proteoglycans and process related impurities such as nucleases and leachables.

The complete downstream processing of LVs is composed of numerous individual unit operations which aim to achieve purification, concentration and stability of the vector material (also reviewed in [180]). Briefly, a typical process, involves sequential purification steps consisting of removing cells and their debris followed by enrichment of vector and the removal of host cell or serum proteins, nucleic acids and lipids. The vector product may be further concentrated prior to exchanging into a suitable formulation buffer for stability and then finally undergoing sterile filtration prior to storage or application (please see Section 6 for whole bioprocess assessment).

### 4.1. Vector Filtration: Initial Clarification

Clarification and sterile filtration processes aim to remove cells and cell debris amongst other large particulate impurities. Clarification typically occurs early in the process, and therefore deals with crude bioprocess material with high load of cells and cell debris and dilute concentration of LVs while sterile filtration occurs in the later stages of the bioprocess and deals with lower concentration of cells or cell debris and higher concentration of LV particles. The initial downstream stages used in clarification are typically microfiltration and/or centrifugation. Cells and bulk particles can be sedimented and removed by low-speed centrifugation [181]. Alternatively, acoustic filters can be used to entrap suspended cells in soundwaves to return to the bioreactor during harvest [162]. Both are followed by microfiltration through a membrane or depth filter. The initial centrifugation acts as a pre-filtration step to prevent premature fouling of the filter. In larger production runs, the capacity of the centrifuge will need to be considered due to capacity considerations. The application of continuous centrifuges, such as disc stack centrifuges, which are prevalent in monoclonal antibody and recombinant protein processing is yet to be seen in LV production given the low-volume processing (litres to hundreds of litres) and the typical batch mode of these processes which need to be balanced with the centrifuge’s high capital cost compared to filtration options.

Filtration techniques, which are effective and scalable, dominate initial clarification, with disposable systems offering simplified cleaning and validation. The use of cascading filters, whereby the feed is filtered with progressively finer filters can prevent premature fouling of the end filter that may result in titre loss due to vector exclusion and extended process times due to reduced flux [182,183]. The choice of filter impacts the efficiency of the clarification step. With membrane filters, the feed passes through a flat or pleated sheet of inert polymer punctuated with pores of a specific diameter according to the membrane’s absolute retention ratings. Depth filters are a class of filters of a sponge-like texture, wherein particles are retained throughout the entire filter bed that is typically composed of polymer, binder and a filtration aid such as diatomaceous earth. For some depth filters, the media bears a charge for electrostatic retention of host cell proteins and DNA [184,185], with diatomaceous earth showing improved feed capacity although at high concentration it can lower LV titre [186]. In some small-scale studies, the use of multiple depth filter media, including cellulose-based and synthetic media (such as polyacrylic fibre with silica filter aids), has shown 90% turbidity reductions whilst maintaining high recovery of non-enveloped vectors, such as simian adenovirus based vectors [187]. In LV applications, depth filters without filter aids such as diatomaceous earth have recovered >95% of titre while reducing >85% HCP at 50 L scale [188]. Typically, most retention ratings are within the microfiltration range from 10 to 0.2 μm in a cascading fashion either applying membranes only or a sequence of a depth filter and a membrane for finer filtration [145,183,189]. At 80–120 nm in diameter, LVs can pass through filters at small retention ratings, although the typical end point of 0.22 μm does see loss in titre [141]. Some examples of available filters for clarification are listed in Table 5.

The performance of the vector is dependent on the retention ratings of the filter, quality of the feed and the load challenge expected at that scale. With highly fouled filters, high transmembrane pressures may result in impurities potentially transferring into the filtrate, likely due to rupture of cells or fragments on the filter’s surface. Clarification is affected by upstream production, with suspension culture necessitating removal of whole cells, whilst adherent culture is likely to be composed of cellular fragments. Moreover, transient transfection for LV production generates different host cell protein profiles compared to stable producer cell lines and can differ depending on envelope protein used [120]. In all cases, some degree of LV loss is to be expected due to adsorption onto the filter or onto a component excluded by the filter, in addition to losses in the filtration system’s hold-up volume. For many clarification processes, flushing the filters with buffer post-process is ideal to dislodge any adsorbed vectors to improve recovery and to clear dead volumes [145].

While commonly applied in concentration and diafiltration (see Section 4.6), tangential flow membrane microfiltration can also be applied in clarification. Any cake layer that builds up on the membrane surface is removed due to the tangential feed flow maintaining flux, as opposed to traditional dead-end filtration modes previously discussed. Recently, a new filter media, a 2–5 μm depth filter from Repligen was used in tangential flow filtration to separate suspension cells and LVs at harvest [191] with reported LV yields of 90%. Such methods can separate suspension cells from LV harvests, allowing the opportunity to recover producer cells for continued culture and sequential harvests.

### 4.2. Vector Filtration: Sterile Filtration

Sterile filtration is typically the final step of the downstream process, wherein the viral vectors, having been purified, concentrated and formulated are passed through a fine filter to remove any adventitious agents such as bacteria or fungi while maintaining LV titres. Titre loss of 30–50% during sterile filtration with a 0.22 μm syringe membrane filter has been reported [145,190,192]. This is partially due to insufficient formulation or poor purification leading to excessive aggregation and vector loss. Due to low vector recoveries and the risk of adventitious agents being present, sterile filtration can be run at an early stage with closed systems or can be revoked wholly, although this would be under close scrutiny of regulatory agencies [97,193].

### 4.3. Non-Chromatographic Purification

The purification of LVs seeks to exploit the differences of the vector and other media components, namely size, density, charge or specificity to a stationary phase. This can be accomplished via numerous methods, with common unit operations being ultracentrifugation and chromatography (see Section 4.5).

Ultracentrifugation is a unit operation that purifies and concentrates vector and is a common application during research and development stages of vector production. This technique has also been broadly used in retroviral, adenoviral and adeno-associated vector purification by pelleting viral particles under large *g* forces [8,194,195]. Due to the large size of the vector compared to free proteins in solution, the vector is pelleted initially, and proteins remain in the supernatant. Alternatively, a gradient is used [196] (typically, sucrose, ficoll or iodixanol is layered in progressively denser layers), and the vector may appear in a specific band, or pellet, with media contaminants in preceding layers, although vesicles or impurities of similar density can be co-sedimented with vector in addition to inhibiting proteoglycans [197,198,199,200]. However, vectors produced by sucrose-gradient display less immunogenic effect in mice, presumably from less serum contamination [201]. The vector pellets can be resuspended, and re-spun for greater purification, with up to four rounds providing relatively clean material [77]. In addition, some envelope proteins may be negatively affected by shear during operation, with VSV-G being particularly resistant [13,76,77]. Further, the particle itself may be damaged by osmolarity of a gradient [202]. Moreover, any gradient composition will likely require removal in sequential processing. High speed centrifuges (10,000× *g*) can also be used in purification, although this may require extended spin times with up to 4 h spin time reported with sucrose [203]. However, centrifugation for the purpose of vector purification can be time-consuming, scale-restricted (linked to capacity of ultracentrifuge unless scale-out is pursued) and labour intensive. Therefore, its application is commonly limited to research, development or early clinical trial phases.

LVs can be precipitated by the addition of excipients into the vector-containing media which can be utilised for concentration purposes. Precipitating agents such as polyethylene glycol (PEG) [65,204], poly-L-lysine [205], a mixture of chondroitin sulphate C and protamine sulphate [206] and calcium phosphate [207] have been employed. In most cases, the vector is incubated with the material before pelleting by centrifugation prior to resuspension and dissociating the precipitates followed by further processing such as TFF or dialysis. Mode of action is a decrease in solubility due to reduced solvent availability leading to vector precipitation or aggregation due to charges. However, the use of precipitating agents may require additional processing for their removal, as well as extended time to fully precipitate the vector particles and to pellet the precipitate with up to 16 h incubation common [65,204,206]. Furthermore, it may be problematic to resuspend and dissociate precipitates, with EDTA treatment required when using calcium phosphate [207]. Despite this, functional recoveries have been reported between 50% and 100% [65,207] across this unit operation.

### 4.4. Nucleic Acid Reduction

Nucleic acid impurities originate from either the plasmid DNA from transfection or host cell DNA from the producer cell lines. Nucleic acids need to be reduced during processing to improve purity and prevent the risk of any deleterious effect in recipient cells or patient, with limits of residual DNA being set out at as <200 bp in length and <10 ng per dose [208,209]. This can be done by the addition of nucleases, such as benzonase, at some point during the process [102,190,210]. Typically, the nuclease is added directly to LV culture in the bioreactor prior to or shortly after harvesting or after clarification. However, due to the greater volumes at these stages, more units of enzyme are required to be effective which incurs costs. An alternative is for the producer or helper cells to secrete the nuclease such as the SecNuc development by Oxford Biomedica [211], wherein a plasmid coding for the nuclease is co-transfected with vector components or the producer cells are co-cultured with nuclease-expressing cells. Otherwise, the nuclease can be added after a concentration step to lower enzyme requirements. Regardless of when the enzymes are added, they must be removed further downstream, and the effect of a high nucleic acid burden should be accounted for, such as co-elution and/or reduced binding capacity in chromatography and the formation of nucleic acid complexes [212,213]. In addition, the composition of the buffer the vector is in must be considered for optimal digestion as some nucleases require magnesium ions, elevated temperature and specific pH ranges to be effective [214] in addition to the required incubation time, which may contribute to vector inactivation. Nucleic acids can also be reduced with careful selection of ion exchange or mixed mode flow-through chromatography (see Section 4.5), or TFF, using stable producer cells (Section 3.2) and whole media replacement in the upstream (Section 3.3).

### 4.5. Chromatographic Purification

Chromatography is a unit operation that separates the components of a mixture via their interactions with stationary and mobile phases. It is a scalable solution for the purification and concentration of particles, in addition to being relatively quick compared to centrifugation and is easily automated for consistent and reliable results. It has seen broad application in the purification of many biologically derived materials such as mAbs, recombinant proteins, industrial enzymes and nucleic acids. Typically, the interactions exploited to resolve feeds are component size in size exclusion chromatography (SEC), charge in ion exchange (IEX), hydrophobicity and affinity to stationary or mobile phases. This can be run in capture and elute mode, or flow-through mode for polishing and refinement. For the purification of LV, multiple types of chromatography can be applied with varying recoveries observed (see Table 6).

**Figure 1:**
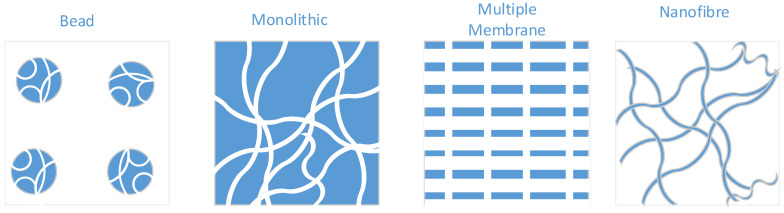
Illustrative examples of chromatography resins in use for lentiviral vector purification with increasing convective mass transfer from left to right.

The development of stationary phases for chromatography has been focused on the purification of small molecules. This has led to the broad application of porous bead based stationary phases to maximise surface area for high dynamic binding capacity. Due to the small sizes of the components, mass transfer is diffusion dominant. This is problematic for viral particles; whereby, due to their size, the pores of beads are too small and therefore their internal surface areas are inaccessible, thus lowering the binding capacity and performance for vector capture [21,218,219,220]. In addition, due to diffusion-based mass transfer, flow rates through the stationary phases tend to be slow, decreasing throughput, which may lead to greater loss of titre due to increased process time. Given these limitations, new stationary phases have been developed that rely on convective based mass transfer for larger particles (see Figure 1). Such phases allow greater flow rates and throughput while maintaining available surface area for binding capacity. The simplest are macroporous stationary phases, such as in monoliths, where numerous channels form a sponge-like structure for feed to flow through. Monoliths are characterised by their large surface area to volume ratios and have been successful in purifying multiple types of virus [221] including LV with different envelopes [119,222]. Alternatively, membrane stationary phases also offer convective mass transfer. These follow a typical flat or woven sheet, possibly stacked on one another to increase capacity, with pores for feed to flow-through and allowing the capture of vector [223,224]. Recently developed are nanofibre spun stationary phases, which offer anion exchange chromatography (AEX) and yield approximately 90% LV recovery [216] as developed by Puridify, now owned by Cytiva (formerly GE Healthcare Life Sciences). Such phases are defined by thin electrospun threads by which the mobile phase can flow past and retention is on the fibres themselves. Such nanofibers have seen utility in the bind-and-elute of LV [216] and adenovirus [225], although commercial capacity is currently focused on the mAb market.

Some hybrid mixed mode chromatography applications have also been developed. While AEX has been shown to be a useful tool for the purification of vector, some impurities are still present in the final elution, particularly DNA and proteoglycans. As a result, some mixed mode stationary phases such as CaptoCore have been developed. These combine size exclusion and AEX chromatography to purify vectors in a negative mode capacity. In this method, the vector is excluded from the internal core of the material which bears AEX ligands. Thus, vector flows through the column, whilst negatively charged DNA or other smaller proteins are captured and removed. This can be applied as a polishing step or contaminant reduction step for vectors and viruses [181,226,227].

#### 4.5.1. Anion Exchange Chromatography

LVs have a net negative charge at neutral pH on their surface [228] due to the composition of the external envelope and their overall isoelectric point, therefore an AEX chromatography with quaternary amines (QA) or diethylaminoethyl (DEAE) ligands are viable options for their capture and elution. With retro or lentiviral vectors, elution tends to be stepped, with the sequential increases in salt concentrations firstly eluting loosely bound components to improve purity before a final high salt buffer elutes the vector. McNally utilised an initial elution of 0.3 M NaCl, before rising to 1.3 M for vector elution, maintaining a pH of 8 throughout Mustang Q AEX [223]. For a weak ligand such as DEAE, this profile can resemble pH 8 and NaCl concentrations of 0.1 and 0.65 M [190]. Stepped elution is particularly effective with vectors, whereby their large size and variable surface composition allow for interaction at multiple sites on the stationary phase and are thus retained to a greater extent compared to individual feed components [228]. This does, however, require greater salt concentrations or pH shift to elute, which necessitates immediate dilution to maintain stability due to osmotic effects [223] or envelope degradation [8,9]. Therefore, the concentration aspect of chromatography is attenuated, and increased buffer consumption for dilution must be considered. In addition, the transient production of LV may give rise to heterologous populations of vectors resulting in differing elution populations that may be attributed to varying compositions of the external envelope [145,229]. Envelope protein free vectors display a lower isoelectric point than envelope positive vectors with varying zeta-potentials which can affect the efficiency of chromatographic purification, likely due to multiple interactions with ligands of the chromatographic stationary phase [228]. Moreover, pre-treating the vector loading material with NaCl has been shown to satisfactorily inhibit bulk protein from binding to AEX material, improving purity [223].

#### 4.5.2. Affinity Chromatography

Affinity chromatography resolves components of a mixture via their specificity to a ligand on the stationary phase. This can be a viable technique that can allow for high purity and concentration of a vector, without the risk of high salt concentrations for elution such as in IEX, thus preserving vector titre during downstream processing. As capture is based on affinity, ideally only vector is retained and eluted, with residual DNA and proteins remaining unbound within the column.

Previously, histidine tags have been expressed on LV envelopes to aid in its purification [215], however yield for this has been low at 46.7% with most presumably remaining attached to the column. Similarly, biotin can be expressed onto the surface of the vector, allowing its capture by immobilised streptavidin and elution by biotin addition [230,231] allowing vector concentration above 4500-fold. This has been further developed by addition of a biotin mimic on a CD8α stalk [167], although, despite high yields at small scale (60%), this declined to 20% on scale up. In addition, a similar method has been applied with Low Affinity Nerve Growth Factor Receptor (LNGFR) being passively incorporated onto the vector’s surface, and thus was able to be captured with an anti-LNGFR antibody on magnetic beads, resulting in excess of 85% recovery, although there was no release from the antibody beads and transduction recovery was only possible by mixing cells with vector-bound beads [232]. Applying affinity tags on the external surface of the vector envelope may also incur some regulatory apprehension if derived from non-mammalian sources, although this may be minimised for ex vivo therapies due to the less stringent requirements at the vector stage. Furthermore, it was observed that retroviral vectors were inhibited when mixed with soluble heparin [233,234,235], leading to the application of heparin affinity chromatography for the purification of LV, whereby heparin and the related heparin sulphate captures LV, leading to recoveries up to 53–61% of vector [157,236].

#### 4.5.3. Size Exclusion Chromatography

Size exclusion chromatography (SEC) is a method to resolve the components of a feed based on size by the flow of material through packed porous beads. Inherently due to their large size in comparison to smaller particulates, LVs can be purified to a high standard with infectious recovery in excess of 70% and purity above 90% [202,237], although high molecular weight contaminants and DNA may remain. In addition, there are applications of SEC to buffer exchange vector into formulation buffer or to desalt after IEX [138]. However, due to the reduced linear flowrate, the overall throughput of the step is low. In addition, SEC is most effective when the feed volume is low, approximately 15% of the bed column, to maintain efficient separation and thus concentration is required prior. As the product of interest is diluted during elution, an additional round of concentration may be required. Ultimately, SEC can be utilised as a polishing step after the bulk of contaminating items are removed in prior stages.

#### 4.5.4. Steric Exclusion Chromatography

Combinations with polyethylene glycol have led to the use of steric exclusion chromatography. The method is selective based on particle size and can be used on viruses as a quicker alternative to SEC. This method follows a similar mechanism as PEG precipitation, with the exception that a hydrophilic stationary phase is present for large particles to coalesce onto. Upon washing the column, the decrease in PEG concentration in the elution buffer releases bound vectors in a size-based selection. Whilst the method has been applied to influenza [238], recoveries at 60% are reported for LV [239].

### 4.6. Concentration and Buffer Exchange by Tangential Flow Filtration

Tangential flow filtration (TFF) is a unit operation often run in ultrafiltration/diafiltration (UF/DF) modalities that allows for volume reduction and buffer exchange in the processing of LV in addition to the removal of low molecular weight impurities while retaining vector particles [240,241]. It is used in various formats (Table 7) to differing parameters (Table 8). Here, tangential ultrafiltration allows volume reduction to concentrate the vector to appropriate titres and simplifies downstream processing with reduced volume feeds. Buffer exchange (diafiltration) often follows a concentration step for optimal replacement buffer consumption efficiency. The choice of buffer that the vector can be exchanged to is important for maintaining vector stability over time and during freeze–thaw (see Section 4.7) in addition to exchanging into optimal chromatography binding buffers or for efficient transduction of recipient cells. Outside of TFF, this can be accomplished by multiple rounds of centrifugation and resuspension in a buffer of choice [77] or SEC methods [237]. However, TFF offers greater autonomy and reduced manual handling while maintaining high vector concentration.

Some TFF systems utilise hollow fibre units, whereas others use cassettes and are composed of varying membranes such as cellulose-based (e.g., mixed cellulose esters) and polyethylene sulphone (PES). Both formats are scalable and applicable solutions for vector concentration and buffer exchange. Both offer high surface area to volume ratios, although flat sheet cassettes can offer higher fluxes than hollow fibres, although at the expense of higher shear rates on vector particles due to their circuitous flow paths and turbulence promoting screens.

Membranes utilising TFF allow the transfer of salts, buffers, and small molecule species across membranes in accordance to their retention rating (typically in kDa). The efficiency of clarification in TFF is related to the retention coefficients of the feed and each individual component. Some groups have utilised TFF as clarification and purification step as an alternative to chromatography, reaching up to 97% recovery [242] or when combined with ultracentrifugation allowing concentration up to 1800-fold [245]. As a result, TFF can be applied in multiple instances such as after chromatography to remove excessive salt from elution buffers, to buffer exchange into formulation buffers, into an optimised binding buffer for capture in chromatography or concentration prior to SEC. Furthermore, if the production of vector were to be intensified, tandem filtration [242] could be employed using hollow fibre membranes to concentrate product in successive hollow fibres of decreasing surface area or applying single-pass tangential flow filtrations, whereby the recirculation loop is absent, and the feed is flowed along a long circuitous membrane path [246]. Although, as of time of writing, no applications for LV have been published, single-pass TFF has seen adoption in mAbs and recombinant protein production [247,248]. As processes mature, the implementation of such unit operations is likely, particularly with perfusion-based bioreactors and stable producer cell lines allowing for optimal continuous production.

Despite this, the parameters of TFF need to be optimised depending on feed, as the flow rate and membrane type can lead to shear damage and loss of vector. In addition, the impact of transmembrane pressure, feed flow rates and the composition of feed affects flux due to fouling which may lead to extended processing times and require larger membranes which entail greater hold up volumes. Vectors can also adsorb or become trapped onto the surface of the membrane particularly if the pore size is of similar magnitude as the vector although larger pores offer better impurity removal and flux performance [145,216,249]. Therefore, membranes within the 100–750 kDa range tend to be employed, whereby larger pore sizes favour flux at the expense of vector adsorption, while lower cut-offs slow flux to maintain vector. Post-run washes can assist in the release of adsorbed vector and optimisation is dependent on the feed at that stage. Moreover, additives to the feed such as sucrose or similar viscosity enhancers may protect the vector from shear [243].

### 4.7. Formulation

The buffer formulation for LV is necessary to maintain the transduction capability of the vector throughout its processing and eventual application. The final formulation will ultimately vary depending on its application. An in vivo therapy for example would require more stringent formulations such as the use of water of injection for safety, compared with vectors for ex vivo applications or for temporary freezing during processing. For many ex vivo therapies, a vector is typically formulated into the cell media for culture in recipient cells [96]; this simplifies the process and no consideration of LV formulation on cell growth is required. However, this does not imply optimal formulation for LV, as the media components may not stabilise the vector satisfactorily or may allow for vector aggregation or adsorption onto containers. This is particularly problematic during freezing, which is required if vector needs to be transported over distances or stored for any amount of time, although the presence of protein and sugars in cell media can be of benefit here.

Typically, buffer excipients act as preservatives for vector formulations. These can feature recombinant proteins, such as human serum albumin, as some protein has been shown to stabilise vectors and sugars such as sucrose or trehalose which can act as cryopreservatives and osmolarity regulators [190]. A buffer species should be utilised to maintain a stable pH (ideally physiological) across a variety of temperatures, and thus species such as HEPES and histidine [250] have been used based on their effective buffering ranges and resistance to pH drift with temperature unlike common buffers such as TRIS which does see pH drift. Despite this, there has been success in the long-term storage of LV with lyophilisation in TRIS or phosphate buffered saline (PBS) with sucrose or trehalose being of benefit [251,252]. The latter demonstrates stability of vector while lyophilised for at least four weeks at 37 °C. Some NaCl may assist in stability, as does the addition of magnesium chloride to prevent aggregation [253]. Additional considerations is the impact of buffers on processing unit operations, for example, negative phosphate ions in common buffer systems interacts with positively charged ligands in AEX chromatography resins.

Kumru et al. [10] examined the effects of multiple excipients on the physical stability of LV, by observation of particle size, morphology and zeta potential where they indicated that the amino acids proline and lysine and the sugars mannose and lactose can minimise vector loss when incubated overnight in glass at 37 °C. Excipients which led to adsorptive losses onto glass were salts (e.g., calcium chloride), reducing agents, chelating agents and cationic peptides. These were also found to negatively affect LV during freeze thaw cycles [10], whereas sugars, polyols and cyclodextrins and certain amino acids such as leucine assisted. However, in this experiment, only physical stability was examined and not transduction capability.

## 5. Vector Characterisation and Quality Control

For applications in human trials, the LV batch should be extensively characterised, and QC tested before release. To maintain safety, the product must remain within a pre-determined specification backed up by suitably validated, precise and repeatable assay protocols. A typical LV batch will require specifications for purity, identity, safety and potency, and these must remain reasonably consistent from batch to batch as required by regulatory agencies, with stringency developing as a potential therapy extends through animal investigations to commercial release. Assays required can be typical and expected of most recombinant processes but can also extend to vector and transgene specific assays. For example, residual DNA can be assayed by Picogreen or quantitative polymerase chain reaction (qPCR), whereas host cell proteins can be analysed by enzyme linked immunosorbent assay (ELISA), SDS-PAGE or any form of total protein quantification such as Bradford, Lowry or bicinchoninic acid colorimetric assay, in addition to standard mycoplasma and endotoxin testing. Additives to the process such as nucleases can be titered by ELISA as can SV40 T-antigen from HEK-293T cells (qPCR for the antigen can signal host cell DNA impurity). Vector specific assays are more complicated due to the nature of the vector particle itself, containing nucleic acids, lipids and proteins, as well as differentiating functional titres and total particle numbers.

Validating quantification methods is essential for the development process and the QA/QC of LV for commercial supply. It is problematic to reach consensus with methodologies due to the variety of transgenes, envelope proteins and recipient cells available across various industrial or academic groups and even inter-group titres vary broadly depending on operator. Despite this, vector quantification is essential when characterising the effectiveness of a production run and is a requirement as a critical quality attribute for regulatory approval. The quantification of LV can broadly be separated into the quantification of various parts of the vector with some degree of crossover, these groupings can be listed as functionality, vector RNA quantification, vector protein, vector enzyme activity and physically counting said particles.

For functionality, the quantification method of choice is the transduction of a known quantity of cells and examining for transgene expression. This method typically requires a titration of the vector of interest across a variety of dilutions and mixing the vector solution and a known quantity of cells together [97]. Polybrene can be added to enhance transduction by minimising electrostatic repulsion between envelope protein and receptor [165]. After a period of time to allow cell expansion and to dilute out any episomal transgene expression, cells are examined for expression, typically by staining with antibodies or affinity-based dyes unless a marker gene is used (often GFP) and analysed with flow cytometry. The transducing units can be determined by knowing the per cent of transduced cells, the volume of vector solution added and the number of cells. However, this does not account for multiple integrations which may arise with high multiplicity of infections and thus titrations must be carried out. Moreover, the risk of overestimating titre due to transgene expression in episomes is apparent, and therefore suitable lengths of time between transduction and cell reading is recommended to dilute out non-integrated transgenes to better reflect long term cell culture for therapies. Furthermore, the total volume, density of cells, availability of cellular receptors and agitation may affect outcomes [254], and thus consistent titres between groups are difficult to compare directly. The transduced cells ideally should be of the same type as the target recipient cell, although typically HEK-293T are used. Moreover, the gating strategy during FACS analysis, the number of transgenes to stain and quality of the stain will need to be considered. In addition, the presence of transduction inhibitors, such as non-functional vector, free floating envelope proteins and proteoglycans, may cause the titre to be under reported in addition to the chance of vector never reaching the cell or available receptor.

A non-staining protocol for functional titre can be carried out via an integration assay where transduced cell genomic DNA (gDNA) is extracted and the provirus is quantified by qPCR and compared to a housekeeping gene. This assay can be unique to the transgene of interest, although a World Health Organization (WHO) standard has been produced for cross group comparison if sequences between the vector transgene and standard are shared [255]. qPCR can quantify multiple integrations although this is not an indicator of transgene functionality. In addition, quality is dependent on gDNA isolation and the lack of DNA contamination from plasmids, host cells and episomal forms [256], and thus expansion time and/or nucleases are required to minimise false positives. Considering the assay is still based on transduction efficiency, the practical applications are in cells where the transgene is difficult to stain for or for legacy sampling of transduced cell gDNA.

Nucleic acid quantification involves the quantification of vector RNA. This method requires the disruption of the vector, isolation of vector RNA, its reverse transcription to complementary DNA and then quantification. Of note, this method does not quantify vector function, and therefore its application is limited to an extent. There is a risk of plasmid DNA inflating the results which necessitates correction with non-reverse transcribed controls or DNase treatment. Furthermore, non-functional but packaged vector may cause over reporting. There is dependence on the efficiency of RNA extraction and its stability, although this can be controlled by a spiked RNA standard. However, its validity may be problematic for process development purposes, whereby varying inhibitors of qPCR or reverse transcriptase in samples may affect results, for example high salt from chromatography elution. qPCR can be further extended with digital droplet qPCR (ddqPCR), wherein individual qPCR reactions are separated by water–oil emulsion droplets at high dilution. By counting the number of positive droplets, the concentration of template can be calculated without a standard curve as responses follow a Poisson distribution. Such technology has been utilised with LV [257], can provide results even if templates are very low in abundance [258] and can be utilised to calculate the number of vector copies in recipient cells [259].

The p24 ELISA assay is a method to quantify the mass of the p24 HIV capsid protein from samples and can be purchased as regular commercial kits. The antibody-based assay can provide quantification of the protein over the course of a day compared to 2–3 days for functional infectivity. In the assay, the vector is disrupted by a detergent before incubation on a plate where either the protein binds by charge onto the plastic or the p24 is captured by a pre-immobilised antibody. An additional primary antibody is incubated and washed away before an enzyme linked secondary antibody is added. After washing, the bound enzyme allows for the colorimetric measurement of a change in a substrate which can be measured by absorbance or fluorescence, which directly corresponds to p24 quantity. This method has seen widespread adoption, with results typically reported as a ratio of the mass of p24 and transducing units (P:I ratio). This links particle mass to functionality and therefore acts as a measure of quality for vector, and even allowing groups to assume LV number by the estimate of $1\times 10^{4}$ particles of LV per pg of p24 [260]. However, p24 kits are reliant on the specificity of their antibodies, and in some cases over report due to the inclusion of non-processed p24 in the form of GAG, vector fragments and inactivated or immature virions.

The measurement of vector enzyme activity can offer an alternative quantification assay for viral proteins. In qPCR-based product enhanced reverse transcription assay (PERT), the vector is titrated and lysed with detergents before mixing with a standard RNA template [261,262]. A thermocycler is set-up with an initial incubation time for reverse transcription to occur, before a temperature rise leads to the enzyme’s inactivation. qPCR is then run to quantify the amount of RNA template converted to DNA, comparing to a known HIV-1 recombinant reverse transcriptase control. As it is dependent on the activity of reverse transcriptase, the assay will be sensitive to inhibitors of reverse transcriptase and may require its stabilisation by inert proteins typically provided in qPCR master mixes. This may appear problematic with processing samples which may have varying ranges of stabilisers or inhibitors. However, the method is rapid, providing results within 2 h and as a result can offer high throughput quantification for multiple samples within a day. The assay can also be more cost-effective than p24 assays due to the lack of specific antibodies and usage of common qPCR mixes.

Another technique is the counting of physical particles. Dynamic light scattering (DLS) is a method whereby the amount of light scattered from a beam when interrupted by a particle is quantified, and, by using the known viscosity of the sample, the hydrodynamic diameter of particles is calculated via the Stokes–Einstein equation. Results can be obtained within 30 min and estimate the mean particle size, polydispersity and a calculation for the particle size distribution. Although this too only determines physical particle numbers and not-functionality, the quality of the data from DLS is progressively unreliable with greater polydispersity, with increased particle numbers causing errant scattering which detracts from the particle of interest. This is problematic with process mixtures which may be reasonably high in polydispersity, in addition to varying viscosities which must be characterised for DLS accuracy. Unless a clean sample is provided, the method is mainly used for average particle sizes in a mixture and determining if a sample is aggregating. Although the DLS technique has been improved with multi-angle dynamic light scattering, which increases the number of detection angles for light scattering and offers more robust results with high polydispersity, accuracy for particle concentration may vary within 50% of a nominal value [263,264].

Alternative methods on similar principles as DLS can be found with nanoparticle tracking analysis methods. Here video clips are recorded through a microscope and the Brownian motion of small particles quantified by a tracking algorithm. Although unable to differentiate LV from other similar sized particles, some newer models allow for the staining and tracking of particles of a specific fluorescence and hence allows for the quantification of specific species. Similarly, tunable resistive pulse sensing detects the size and number of particles passing through a small pore and have been used with LV [265]. Other physical particle-based quantification can be accomplished by counting particles in electron microscopy with a negative stain, modern systems, e.g. the benchtop MiniTem from Vironova, offer automated electron microscopy and analysis [266]. In addition, the fluorescence of tagged LV can be compared with fluorescent beads in confocal microscopy. Both methods can be very time consuming to complete and insufficient for large numbers of samples. A recent addition, which utilises high-performance liquid chromatography (HPLC) with AEX resins, can elute bands of vector, and, based on their fluorescence, estimate the number of vector particles within $10^{8}$ to $10^{10}$ total particles per mL range [267]. This method has been used to differentiate DNA from vector and is comparable to ELISA and ddPCR and allows for the application of various samples from differing aspects of the process within 6.5 min. Although this method only quantifies vector particles and does not display functional units, such rapid and high throughput analytical considerations are of strong value for process development.

## 6. Whole-Bioprocess Assessment of LV Production

**Figure 2:**
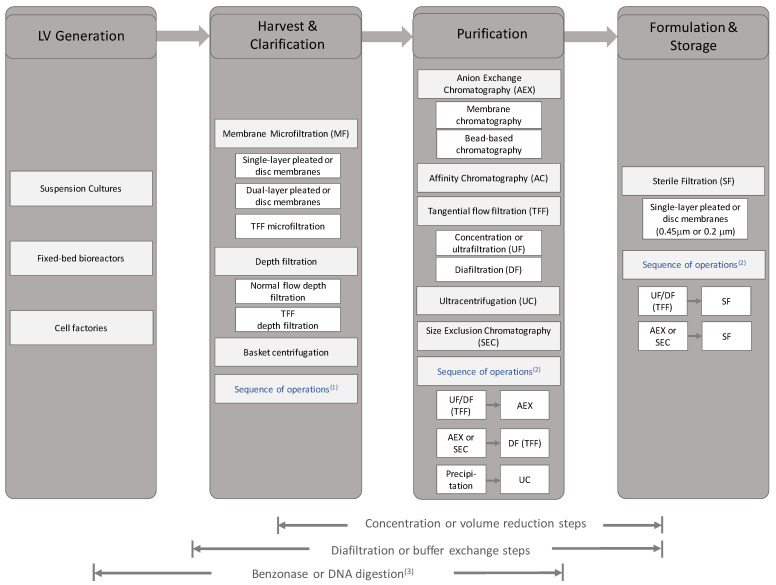
Bioprocess options in the production of lentiviral vectors. ${}^{\left(1\right)}$ Some studies have shown sequence of membrane filtration of different pore sizes or inclusion of low-speed centrifugation prior [145,183,189]. Sequence of filtration processes would be an option depending on scale and cell density and product and impurity profile. ${}^{\left(2\right)}$ These are examples of sequences of operations used in pre-clinical and clinical investigations [145,268,269]. ${}^{\left(3\right)}$ Benzonase may be added at a variety of steps within the downstream process.

Figure 2 illustrates the different process options available for the manufacture of lentiviral vectors. There are process options which are only useful at small scale or for applications where low number of doses are required (ultracentrifugation, SEC and basket centrifugation) while mostly are scalable (e.g., filtration options, chromatography, etc). The final sequence of operations depends on the scale of application of the LV product, the technology used for LV generation, LV titres and the desired product and impurity profile. In this regard, the actual point in which certain operations need to be applied (e.g., concentration, diafiltration, DNA digestion, etc) will depend on these factors. For example, the application of benzonase, or similar products, for DNA digestion may be performed anywhere from the LV generation step [211], as part of the clarification step [97] or before or after chromatography [190].

In their review paper, McCarron et al. [270] provided an overview of the challenges of scaling-up lentiviral vector production. These are briefly summarised in Table 9. Among the challenges in LV production, bioprocess understanding is most relevant in addressing the low recoveries and loss of vector functionality. This starts with the understanding of the application of the final product, which define the final scale of operation and, the product and process specifications. In addition, determining the relevant parameters which impact the performance of process options will be important in the optimal selection of these options and their operation. Screening studies can provide crucial bioprocess information such as the level of transmembrane pressures [65] or crossflow rate [145] in TFF operations, column flow rate in AEX chromatography [145] or the right molecular weight cut-off (MWCO) or membrane material in TFF operation [65,145,243,244]. It is also important to determine the impact of using frozen-thawed materials in process development of unformulated LV products (e.g., [190,216]), as opposed to using fresh material, as this step may have a huge influence on the process performance rather than as result of the bioprocess operation itself.

Defining the steps in the bioprocess sequence of LV production requires a whole-bioprocess analysis due to the interaction among the different operations. For example, concentration and buffer exchange (TFF) followed by AEX chromatography produced an LV product that when used in transduction was not toxic to cells, despite the lower overall yield compared to just using a TFF step [65]. In another example, TFF operation, whether on its own or combined with ultracentrifugation, resulted in an LV product with higher functional titre not seen when this step was removed [245]. Finally, the location of DNA digestion within the process sequence [145] or the location of the TFF step [243] may be used to improve the following AEX chromatography.

The increased interest in viral vectored cell and gene therapies pushes the boundaries of what is currently done in bioprocessing. For larger scale LV production, the role of process shear needs to be investigated as large-scale production means using larger pumps or running pumps at higher flowrate and therefore, potentially higher shear rates. Furthermore, higher productivity requirements also mean increased flux requirements to shorten the time during TFF operation. This impacts LV production in several ways: the shorter time may be beneficial for LV stability while the increased flux may mean the need to run at higher crossflow rates [145] or higher transmembrane pressure [65]; both may result in exposure to high process shear [271,272]. Viral vectors in general are known to be fragile and therefore sensitive to shear. For example, Valkama et al. [145] mentioned that an increased recirculating flowrate, by-passing a column, resulted in the 20% loss of infective LVs. However, an early analysis of work in our lab found that some pseudotyped LVs have high recoveries even after exposure to very high process shear using ultra scale-down (USD) (unpublished data). This demonstrates that process shear may have different effects on different lentiviral vectors and that the design of bioprocess operations (e.g., TFF) should account for these in order to increase productivity and meet requirements at larger-scale manufacturing. We previously demonstrated the use of ultra scale-down devices to predict a larger scale TFF operation to produce monoclonal antibodies [273]. This larger-scale equipment is of similar type to that used in LV TFF processing [140,145]. Ultra scale-down approaches have also been used to evaluate other unit operations [272]. USD enables whole-bioprocess assessment because of the small amount of material required to perform the analysis. Lastly, as part of the whole-bioprocess analysis of the production of LVs, incorporating a process economic analysis would be beneficial as it could demonstrate the economic viability of bioprocess options [27].

## 7. Conclusions

We reviewed the basic unit operations, whole bioprocess options and other current developments in the bioprocessing of lentiviral vectors. The demand for LVs will remain high in the foreseeable future as the therapeutic benefits of cell and gene therapy are realised and transferred into the clinic with new applications being explored (e.g., as viral vaccine vectors [274]). Although current manufacturing capacity for LVs is low globally, and LV bioprocessing requires optimisation, efforts are apparent which are improving yields and recoveries. Such developments will lead to greater implementation of gene transfer agents to improve therapeutic outcomes. The fundamental understanding of the bioprocess requirements of lentiviral vectors is key in ensuring the translation of LV products from clinical development to use by patients. TFF and AEX chromatography are front-runners as unit operations of choice for scalable LV bioprocessing as does microfiltration. From what we already know of these operations, the solution environment (i.e., buffers, additives, excipients, etc.) as well as the solid-phase materials (e.g., membranes, resins or fibres) will have important contributions during processing of different pseudotyped LVs. The determination of key operational parameters and process conditions will be an essential activity in process development, along with a whole bioprocess assessment. This should lead to obtaining high LV concentrations and yields with minimal impurities in the LV product.

## Figures and Tables

**Table 1:** Common impurities within the processing of lentiviral vectors.

Product Impurities	Process Impurities
Non-functional vector (broken, immature, insufficient envelope protein or non-packaged)	Host cell proteins, lipids, DNA and debris
Free Vector Envelopes	Plasmid DNA
Viral aggregates	Transfection Reagents
Free vector components	Expression Enhancers/Inducing Agents
Proteoglycans	Buffers
	Salts
	Nucleases
	Culture Leachables
	Serum derived protein, amino acids, lipids and salts
	Media derived sugars, buffers and salts

**Table 2:** Examples of envelope proteins that have been pseudotyped with lentiviral vectors.

Pseudotyped Envelope Protein	Virus Origin	Receptor	Cell Target	Reported Titre, Transgene, Assay Cell Target, Production Mode	Ref.
RD114	Feline endogenous virus	ASCT-2	T, B Cells	$1.3\times 10^{6}$ TU/mL, GFP, HeLa, transient [30]	[30,31,32]
RDPro	Feline endogenous virus	ASCT-2	T, B Cells	$4\times 10^{5}$ TU/mL, GFP, 293T, transient [33], $10^{6}$ TU/mL, GFP, 293T, stable [7]	[7,31,33,34]
RD114-TR	Feline endogenous virus	ASCT-2	T, B Cells	$1.5\times 10^{6}$ TU/mL, GFP, 293T, transient [33]	[31,33,35]
VSV-G	Vesicular Stomatitis Virus	LDL-R	Near ubiquity	$2.3\times 10^{7}$ TU/mL, GFP, HeLa, transient [30]	[13,30]
Cocal-G	Cocal	LDL-R	Near ubiquity	$10^{6}$ TU/mL, GFP, HT1080, transient [36], $10^{6}$ TU/mL, GFP, HT1080, stable [36]. $8.2\times 10^{4}$ TU/mL, GFP, 293T, stable [37]	[36,37]
GALV-TR	Gibbon ape leukaemia virus	SLC20A1 (GLVR1)	Hematopoietic Cells	$10^{6}$ TU/mL, GFP, 293T, transient [33]	[33,38,39,40]
BaEV	Baboon ape leukaemia virus	ASCT-2, ASCT-1	Hematopoietic Cells	$10^{2}$ IU/mL, GFP, 293T, transient [41]	[31,41,42]
BaEV-TR	Baboon ape leukaemia virus	ASCT-2, ASCT-1	Hematopoietic Cells	$10^{5}$ IU/mL, GFP, 293T, transient [41]	[31,41,42]
BaEVRLess	Baboon ape leukaemia virus	ASCT-2, ASCT-1	Hematopoietic Cells	$10^{6}$ IU/mL, GFP, 293T, transient [41]	[31,41,42]
H/F	Measles	SLAM, CD46	Lymphocyte	$4.7\times 10^{6}$ TU/mL (100× concentrated), GFP, 293T, transient [43]	[43,44,45]
G/F	Nipah	EphrinB2, EphrinB4	Hematopoietic Stem Cells, endothelial cells	$10^{4}$ to $10^{5}$ TU/mL (truncation-dependent), GFP, 293, transient [46]	[46,47]
RabV	Rabies	p75NTR, CD56, nAchR	Neuronal	$10^{7}$ LacZ-forming units/mL (ultracentrifuge), LacZ, 293T, transient [48]	[48,49,50,51,52]
MOKV	Mokola	Likely similar to RabV	Neuronal	$10^{6}$ CFU/μg p24, β-galactosidase, HOS, transient [53]. $3\times 10^{6}$ TU/mL GFP, 293T, transient [54]	[53,54,55,56]
SV	Sindbis	Integrin, heparin sulphate	67LR, NRAMP2	$10^{5}$ TU/mL, GFP, 293T, transient [57]	[57,58,59,60]
PRYV-G	Piry	Unknown		$5\times 10^{8}$–$1.4\times 10^{9}$ TU/mL (ultracentrifuged 500–1000×), GFP 293T, transient, [61]	[37,61]
CNV-G	Chandipura	Unknown	Neuroblastoma Cells	6.9–9.1$\times 10^{8}$ TU/mL (ultracentrifuged 500–1000×), GFP 293T, transient, [61,61]	
Ampho	Murine Leukaemia Virus	SLC20A2 (GLVR2)	Hematopoietic Cells	$1.7\times 10^{6}$ TU/mL, GFP, HeLa, transient [30]. $4\times 10^{6}$ CFU/mL, β-galactosidase, 293T, transient, [40]	[30,40,62]
F/HN	Sendai	ASGP-R	Hepatocytes, T lymphocytes	$1.3\times 10^{6}$ TU/mL, β-galactosidase, HT1080, transient [11]	[11,63,64]
△GP Jenv	Jaagsiekte Sheep Retrovirus	hyaluronidase 2	Lung Epithelial Cells	$1.1\times 10^{6}$ TU/mL, GFP, 293T, transient [65]	[65,66,67]

**Table 3:** Stable and inducible cell lines for Lentiviral vector production.

Cell Line	Inducible	Vector Generation	Envelope	Transgene	Reported Titre	Adherent or Suspension	Notes	Ref.
LentiPro26	N/A	3rd Generation	MLV Amphotropic Envelope	GFP	$10^{6}$ TU/mL/Day	Adherent	Mutated HIV-1 protease for less cytotoxic activity.	[121]
WinPac	N/A	3rd Generation	RDPro	GFP	$10^{6}$ TU/mL	Adherent	Retroviral Tagging and Recombinase Mediated Cassette Exchange for high Gag-Pol expression.	[7]
STAR	N/A	2nd Generation	MLV Amphotropic Envelope	GFP	$10^{7}$ IU/mL	Adherent	HIV-1 Gag-Pol delivered by MLV vector.	[34,81]
STAR	N/A	2nd Generation	RDPro	GFP	$10^{7}$ IU/mL	Adherent	HIV-1 Gag-Pol delivered by MLV vector.	[34,81]
RD2-MolPack	N/A	2nd Generation	RD114-TF	GFP	$10^{6}$ TU/mL	Adherent	RD114 fused to cytoplasmic tail of MLV-ampho 4070. Baculo-AAV integration of Gag-Pro-Pol and Rev.	[122]
RD3-MolPack	N/A	3rd Generation	RD114-TF	GFP	$3.7\times 10^{5}$ TU/mL	Adherent	RD114 fused to cytoplasmic tail of MLV-ampho 4070. Baculo-aav integration of Gag-Pro-Pol and rev.	[123]
GPRG-TL20-GFP	TET-OFF	3rd Generation	VSV-G	GFP	$10^{7}$ TU/mL	Adherent	Concatemeric array transfection technique for SIN vector genome	[124]
GPRG-TL20-IL2RG	TET-OFF	3rd Generation	VSV-G	IL2RG	$5\times 10^{7}$ TU/mL	Adherent	Concatemeric array transfection technique for SIN vector genome	[124]
EF1αhWASp	TET-OFF	3rd Generation	VSV-G	hWASp	$5\times 10^{7}$ IU/mL	Adherent	Based on GPRG	[125]
293SF-PacLV	TET-ON, Cumate	3rd Generation	VSV-G	GFP	$3.4\times 10^{7}$ TU/mL/day	Suspension	Serum Free	[126]

**Table 4:** Examples of upstream cell culturing for the production of lentviral vector.

Supplier	Model	Volume/Growing Area	Cell Growth	Vessel Type	Ref.
Corning	CellCube	8500 to 85,000 cm${}^{2}$; 2.6–8 L	Adherent	Multilayer and Perfusion	[137]
Corning	CellSTACK	636–25,440 cm${}^{2}$ (1–40 stacks)	Adherent	Multilayer	[97]
Corning	Hyperflask	1720 cm${}^{2}$; 500 mL	Adherent	Multilayer	[138]
Corning	HyperStack	6000–18,000 cm${}^{2}$ (12–36 layers)	Adherent	Multilayer	[139]
Cytiva *	WAVE	10–200 L	Suspension	Rocking Platform	[140]
Cytiva *	Xcellerex XDR	4.5–2000 L	Suspension	Stirred Tank	[141]
Eppendorf	BioBLU 5p	Up to 180,000 cm${}^{2}$	Adherent	Fixed Bed	[142]
Pall	Allegro STR	20–2000 L	Suspension	Stirred Tank	[143]
Pall	iCELLIS	0.53–4 m${}^{2}$; 1 L (Nano). 66–500 m${}^{2}$; 120 L (500 Model)	Adherent	Fixed Bed	[144,145]
Sartorius	AMBR	10–250 mL	Suspension	Stirred Tank	[146]
Sartorius	BioStat	1–2000 L	Suspension	Stirred Tank	[147]
ThermoFisher	NUNC Cell Factories	632–25,280 cm${}^{2}$; 1–40 Layers	Adherent	Multilayer	[148]
Univercells	Scale-X	2.4 m${}^{2}$ (Hydro); 10–30 m${}^{2}$ (Carbo); 600 m${}^{2}$ (Nitro)	Adherent	Fixed Bed	[149]

* Formerly GE Healthcare Life Sciences.

**Table 5:** Examples of filtration units for initial clarification of lentiviral vectors.

Chemistry	Cell Culture	Constant Flux or Constant Pressure	Load Challenge	Vector Recovery	Ref
PES	HEK-293T adherent	Constant Flux-(1200 LMH)	112 L/m${}^{2}$	91 ± 6%	[190]
PES	HEK-293T adherent	Constant Flux-(220 LMH) (small scale); (146 LMH) (large scale)	290 kg/m${}^{2}$ * (small scale); 215 kg/m${}^{2}$ * (first scale-up); 232 kg/m${}^{2}$ * (second scale-up)		[145]
Cellulose Acetate	HEK-293T adherent	Constant Flux-(220 LMH) (small scale); (146 LMH) (large scale)	290 kg/m${}^{2}$ * (small scale); 215 kg/m${}^{2}$ * (first scale-up); 232 kg/m${}^{2}$ * (second scale-up)	75–90% functional particle, 100% total particles	[145]
PES	HEK-293T Adherent	Constant Pressure- (0.5 barg)	≈175 L/m${}^{2}$ *	≈71% *	[183]
PVDF	HEK-293T Adherent	Constant Pressure- (0.5 barg)	≈360 L/m${}^{2}$ *	≈78% *	[183]
Nylon 66	HEK-293T Adherent	Constant Pressure- (0.5 barg)	≈390 L/m${}^{2}$ *	≈77% *	[183]
Resin-bonded GF	HEK-293T Adherent	Constant Pressure- (0.5 barg)	≈1850 L/m${}^{2}$ *	≈100% *	[183]
Polypropylene	HEK-293Tsa (suspension)	Constant Flux-(150 to 75 LMH)	90 L/m${}^{2}$	>70% physical titre (ELISA); >95% TU/mL	[188]

* Calculated from data in text. PES, Polyethersulphone; PVDF, Polyvinylidene fluoride.

**Table 6:** Example of chromatographic operations in lentiviral vector purification.

Unit Name	Mode	Stationary Phase	Ligand Chemistry	Column Volume	Equilibration Buffer (Composition and CV)	Wash Buffer (Composition and CV)	Elution Buffer (Composition and CV)	Elution Type	Feed (Composition and CV)	Envelope Protein	Vector Recovery	Ref.
BIA CIM-IDA-NI${}^{2+}$	Affinity	Monolith	IMAC (with Histidine Tagged LV)	0.34 mL	5 CV PBS supplemented with 10 mM imidazole	30CV, 3 mM imidazole, PBS	150 mM imidazole	Isocratic	Up to 117 CV * DMEM, 10% FBS, 10 mM imidazole	VSV-G	69% ****	[215]
CIM DEAE	AEX	Monolith	DEAE	8 mL	10 mM Tris (pH 8)	10 mM Tris (pH 8)	Up to 1.5 M NaCl, 10 mM Tris (pH 8)	Gradient	18.75 CV * (150 mL)	VSV-G	65%	[119]
CIM DEAE	AEX	Monolith	DEAE	8 mL	10 mM Tris (pH 8)	10 mM Tris (pH 8)	0.1 M, 0.65 M NaCl (10 mM Tris (pH 8))	Stepwise	DMEM, 10% FBS	VSV-G	80%	[190]
CIM DEAE	AEX	Monolith	DEAE	8 mL	10 mM Tris (pH 8)	10 mM Tris (pH 8)	0.1 M, 0.65 M NaCl, 50 mM HEPES (pH 8)	Stepwise	DMEM, 10% FBS	VSV-G	71%	[190]
CIM DEAE	AEX	Monolith	DEAE	8 mL	10 mM Tris (pH 8)	10 mM Tris (pH 8)	0.1 M, 0.65 M NaCl (10 mM PBS	Stepwise	DMEM, 10% FBS	VSV-G	74%	[190]
CIMac Streptavidin	Affinity	Monolith	Streptavidin–Biotin Mimic	0.1 mL	50 CV PBS, 500 CV DMEM, 50CV PBS	2 × 300 CV, PBS	150 CV X-Vivo15 media supplemented 15 mM Biotin, 0.5% BSA	Isocratic	500 CV, serum-free DMEM	RDPro	20%	[167]
Fibro (Prototype)	AEX	Nanofiber	RC QA	0.1 mL	PBS	PBS	0–50% elution buffer over 100 CV, 60 CV step to 100%. PBS, and PBS +2 M NaCl	Gradient/Step	4000 CV; DMEM, 10% FBS	RDPro	90%	[216]
FractoGel	Affinity	Bead	Heparin	1 mL	150 mM NaCl. 20 mM Tris (pH 7.5)	15 CV; 150 mM NaCl	13 CV; 350 mM NaCl	Gradient	35 CV; Freestyle	VSV-G	53%	[157]
HiTrap Q	AEX	Bead	Quaternary Amine	1 mL	10 CV; 100 mM Tris (pH 7.5)		100 mM Tris (pH 7.5) + 1 M NaCl	Gradient	1 CV; 200× concentration LV in equilibration buffer	VSV-G **	53% *** after ultracentrifugation and desalting	[181]
Mustang Q	AEX	Membrane	Quaternary Amine	60 mL	40 CV, 25 mM Tris-HCl (pH 8)	40 CV, equilibration buffer	20 mL 25 mM Tris-HCl (pH 8), 1.2 M NaCl	Isocratic	100 CV, DMEM, 10% FBS, 1% sodium pyruvate	VSV-G	Final titre approximately $10^{8}$ to $10^{9}$ TU in 150 mL from 6 L non-concentrated	[139]
Mustang Q	AEX	Membrane	Quaternary Amine	0.18 mL		11 CV *, 25 mM Tris-HCl (pH 8), 0.6 M NaCl	0.3 M to 1.5 M NaCl in 25 mM Tris- HCl (pH 8)	Stepwise	2777 CV *. DMEM, 10% FBS, 1% GlutaMAX, 1% Pen/Strep adjusted to 25 mM Tris-HCl (pH 8), 0.6 M NaCl	VSV-G	76%	[138]
Mustang Q	AEX	Membrane	Quaternary Amine	0.8 mL	12.5 CV * 150 mM NaCl, 25 mM Tris-HCl (pH 7.4)	12.5 CV * 150 mM NaCl, 25 mM Tris-HCl (pH 7.4)	1M NaCl, 25 mM Tris-HCl (pH 7.4)	Stepwise	675 CV; DMEM, 10% FBS **	Measles M/V	80%	[217]
Mustang Q	AEX	Membrane	Quaternary Amine	0.8 mL	12.5 CV * 150 mM NaCl, 25 mM Tris-HCl (pH 7.4)	12.5 CV * 150 mM NaCl, 25 mM Tris-HCl (pH 7.4)	0.2 to 0.4 NaCl, 25 mM Tris-HCl (pH 7.4)	Gradient	675 CV; DMEM, 10% FBS **	Measles M/V	65%	[217]
Sartobind D	AEX	Membrane	DEAE	2 mL	10 mM Tris (pH 8)	10 mM Tris (pH 8)	Up to 1.5 M NaCl, 10 mM Tris (pH 8)	Gradient	75 CV (150mL)	VSV-G	29%	[119]
Sartobind D	AEX	Membrane	DEAE	2.1 mL	10 mM Tris (pH 8)	10 mM Tris (pH 8)	0.1 M, 0.65 M NaCl, 50 mM Tris (pH 8)	Stepwise	DMEM, 10% FBS	VSV-G	28%	[190]
Sartobind Q	AEX	Membrane	Quaternary Amine	400 mL	50 mM HEPES, 300 mM NaCl (pH 7.5)	50 mM HEPES, 300 mM NaCl (pH 7.5)	12, 30, 45% elution. 1.5 M NaCl, 50 mM HEPES (pH 7.5)	Stepwise	11 kg concentrated and Buffer exchanged	VSV-G	33.1%	[145]

* Calculated from text; ** assumption based on text; *** recovery based on integration copy numbers; **** elution efficiency: TU eluted/captured.

**Table 7:** Examples of tangential flow filtration operations and membrane materials in lentiviral vector processing.

Pseudotype	Step Before TFF	TFF Operation	Membrane Type. (MWCO, Total Area), Brand, Supplier	Membrane Format (TFF System)	Ref.
VSV-G	Clarification	UF (20× CF). DF (10 DV)	Cellulose. Ultracel type V (100 kDA or 300 kDa, 0.1 m${}^{2}$), Pellicon 2, Merck Millipore	Flat Sheet Cassette (n.m.)	[145]
VSV-G	Clarification	UF/DF	PES. Biomax type V (100 kDA, 0.1 m${}^{2}$), Pellicon 2, Merck Millipore	Flat Sheet Cassette (n.m.)	[145]
VSV-G	Clarification	UF/DF	Cellulose. Hydrosart (100 kDA, 0.11 m${}^{2}$), Sartocon Slice, Sartorius	Flat Sheet Cassette (AKTA crossflow)	[145]
VSV-G	Clarification	UF/DF	Cellulose. Hydrosart (100 kDA, 0.11 m${}^{2}$), Sartocon Slice, Sartorius	Flat Sheet Cassette (Cogent M)	[145]
VSV-G	Clarification	UF (13–18× CF) DF (9–10 DV)	Cellulose. Hydrosart (100 kDA, 5 × 0.6 m${}^{2}$ or 1 × 3 m${}^{2}$ + 2 × 0.6m${}^{2}$) Sartocon or Sartocube, Sartorius	Flat Sheet Cassette (Mobius)	[145]
VSV-G	Clarification	UF (110× CF) DF (20 DV) (Tandem TFF—1 of 2)	Membrane type (n.m.). 320 fibres (0.5 mm internal diameter) with a total surface area of 615 cm${}^{2}$ and a 500 kDa cut-off	Hollow Fibre. (KrosFlo Research II TFF System)	[242]
VSV-G	TFF (Tandem TFF—1 of 2)	UF only (50× CF) (Tandem TFF—2 of 2)	Membrane type (n.m.). 12 fibres (0.5 mm internal diameter) with a total surface area of 40 cm${}^{2}$ and a 500 kDa cut-off	Hollow Fibre. (KrosFlo Research II TFF System)	[242]
VSV-G	Clarification	UF (10–22× CF) DF (10–11 DV)	36 mPES fibres (0.5 mm diameter, 20cm) with a total surface area of 115 cm${}^{2}$ and 500 kDA cut-off	Hollow Fibre. (KrosFlo Research II TFF System, Spectrum Labs, Repligen)	[243]
VSV-G	Clarification	UF (10-22x CF) DF (10-11 DV)	Cytiva (former GE Healthcare) PES (1 mm diameter, 30 cm) with a total surface area of 110 cm${}^{2}$ and 750 kDA cut-off	Hollow Fibre. (KrosFlo Research II TFF System, Spectrum Labs, Repligen)	[243]
GalV-TR	Clarification	UF (10–22× CF) DF (10–11 DV)	Cytiva (former GE Healthcare) PES (1 mm dia., 30 cm) with a total surface area of 110 cm${}^{2}$ and 750 kDA cut-off	Hollow Fibre. (KrosFlo Research II TFF System, Spectrum Labs, Repligen)	[243]
VSV-G.	Clarification	UF only	Omega PES (200 cm${}^{2}$). 50 kDA, 100 kDA, 300 kDA	Flat sheet cassette. (Centramate, Pall)	[65]
VSV-G	Clarification	UF only	Omega PES (200 cm${}^{2}$). 50 kDA, 100 kDA, 300 kDA	Flat sheet cassette. (Centramate, Pall)	[65]
ΔGP JenV	Clarification	UF only	Omega PES (50 kDa, 200 cm${}^{2}$).	Flat sheet cassette. (Centramate, Pall)	[65]
ΔGP JenV.	Clarification	UF only	Omega PES (50 kDa, 200 cm${}^{2}$).	Flat sheet cassette. (Centramate, Pall)	[65]
RDPro	Clarification	DF only	36 mPES fibres (0.5 mm diameter, 20 cm) with a total surface area of 115 cm${}^{2}$ and 500 kDA cut-off	Hollow Fibre. (KrosFlo Research II TFF System)	[216]
VSV-G	Chromatography/ Benzonase	UF only (30–40× CF)	Membrane type (n.m.). (100 kDA, 50 cm${}^{2}$)	Hollow fibre. (Cytiva)	[244]
VSV-G	Chromatography/ Benzonase	UF only (30–40× CF)	Membrane type (n.m.). VivaFlow (100 kDA, 50 cm${}^{2}$)	Flat sheet cassette. (Vivaflow, Sartorius)	[244]
VSV-G	Chromatography/ Dilution	DF/UF/DF	Membrane type (n.m.). VivaFlow (100 kDA, 50 cm${}^{2}$)	Flat sheet cassette. (Vivaflow, Sartorius)	[98]
BaEV-R-less	Chromatography/ Dilution	DF/UF/DF	Membrane type (n.m.). VivaFlow50 (100 kDA, 50 cm${}^{2}$)	Flat sheet cassette. (Vivaflow, Sartorius)	[98]
VSV-G	Chromatography	UF (10× CF). DF (2 DV)	Membrane type (n.m.). Pellicon-2 (500 kDA, 0.1 m${}^{2}$)	Flat Sheet Cassette. (Pellicon-2 Mini, Merck Millipore)	[140]
VSV-G	Chromatography	UF Only	Membrane type (n.m.). VivaFlow (100 kDA, 50 cm${}^{2}$)	Flat sheet cassette. (Vivaflow, Sartorius)	[190]
VSV-G	Chromatography	UF Only	Membrane type (n.m.). (100 kDA, 50 cm${}^{2}$)	Hollow fibre. (Cytiva)	[96]

**Table 8:** Examples of tangential flow filtration operating conditions in lentiviral vector processing.

Pseudotype	TFF Mode: Permeate Flux Control (Flux, TMP${}_{Max}$) or TMP Control *	Notes	Ref.
VSV-G	Constant flux (n.m., 0.05–0.1 bar)	59–74 L/m${}^{2}$ feed load. DF buffer 50 mM HEPES + 300 mM NaCl (pH 7.5)	[145]
VSV-G	Constant flux (n.m., 0.10 bar < TMP${}_{max}$ < 0.2 bar)	30–35 L/m${}^{2}$ feed load during concentration. DF buffer 50 mM HEPES + 300 mM NaCl (pH 7.5)	[145]
VSV-G	Constant flux. (10 LMH, 0.06 bar)	10–70 kg/m${}^{2}$ feed load. 3 L/h Feed flowrate ** (64% crossflow). DF buffer 50 mM HEPES + 300 mM NaCl (pH 7.5)	[145]
VSV-G	Constant flux. (24–100 LMH, 0.2–0.5 bar)	50–120 kg/m${}^{2}$. Typically 60 kg/m${}^{2}$ feed load. 20–110 L/h Feed flowrate ** (88–90% crossflow). DF buffer 50 mM HEPES + 300 mM NaCl (pH 7.5)	[145]
VSV-G	Constant flux (13–14 LMH, 0.1–0.2 bar)	42–43 kg/m${}^{2}$ feed load. 350–496 L/h Feed flowrate ** (89% crossflow). 70% recovery, >70% dsDNA clearance. DF buffer 50 mM HEPES + 300 mM NaCl (pH 7.5)	[145]
VSV-G	Constant TMP (feed control, <6 psi (0.4 bar))	DF buffer is a 1L mixture of Dulbecco’s Phosphate Buffered Saline with fetal calf serum (FCS). >97% recovery	[242]
VSV-G	Constant TMP (feed control at <9 psi, (0.6 bar))	>97% Recovery.	[242]
VSV-G	Constant TMP (feed control at 7 psi, 0.5 bar)	40% recovery. Membranes pre-treated with DMEM and FBS. Performed at 4 °C. Feed flowrate, at 40 mL/min	[243]
VSV-G	Constant TMP (feed control at 7 psi, 0.5 bar)	Up to 100% recovery depending on buffer composition (pH, salt, sucrose, etc). Membranes pre-treated with DMEM and FBS. Performed at 4 °C. Feed flowrate, at 40 mL/min	[243]
GalV-TR	Constant TMP (feed control at 7 psi, 0.5 bar)	Up to 80% recovery depending on buffer composition (pH, salt, sucrose, etc). Membranes pre-treated with DMEM and FBS. Performed at 4 °C. Feed flowrate, at 40 mL/min	[243]
VSV-G.	Constant TMP (feed control at 5 psi (0.3 bar), retentate backpressure 0–5 psi)	“Low TMP”. Recoveries are 3% (50 kDA), 21% (100 kDa), and 11% (300kDa) at 0.3 bar TMP	[65]
VSV-G	Constant TMP (feed control at 15 psi (1 bar), retentate backpressure 10 psi)	“high TMP” (0.8 bar). Recoveries are 100% (50 kDa), 91% (100 kDa), and 81% (300 kDa). Overall recovery using 50 kDa is 99.6%	[65]
ΔGP JenV	Constant TMP (feed control at 5 psi (0.3 bar)	“Low TMP” (0.3bar TMP). 99% recovery	[65]
ΔGP JenV.	Constant TMP (feed control at 15 psi (1 bar), retentate backpressure 10 psi)	“high TMP” (0.8 bar). 98% recovery. Overall recovery using TFF is higher than overall recovery using ultracentrifugation	[65]
RDPro	Constant TMP = 1 psig (feed control at 20–30 mL/min)	20 mM Tris (pH 7.4)	[216]
VSV-G	Constant TMP (feed flow at 75 mL/min, 0.3 bar TMP)	Benzonase addition and 1:1 dilution immediately performed after elution (i.e., before TFF). Measured permeate flux is 30–35 LMH	[244]
VSV-G	Constant TMP (feed control at 1–1.5 bar)	Benzonase addition and 1:1 dilution immediately performed after elution (i.e., before TFF). Measured permeate flow is 5 mL/min	[244]
VSV-G	n.m.	From suspension cells. 40–70% overall recovery. 50-fold concentration from harvest	[98]
BaEV-R-less	n.m.	From suspension cells. 30–36% overall recovery. 50-fold concentration from harvest	[98]
VSV-G	n.m.		[140]
VSV-G	Constant TMP (feed control at 1 bar)	Feed flowrate at 7 mL/min. 72% recovery. Membranes pre-treated with human serum albumin (HSA)	[190]
VSV-G	n.m.	Overall yield: 16–24%	[96]

n.m., not mentioned in reference; * pressure control—feed control is a form of Transmembrane Pressure (TMP) control if the difference between inlet and outlet (retentate) pressure is maintained; ** calculated from data in the reference.

**Table 9:** Known challenges in LV Production.

No.	Challenge *	Solution **
1	Reliance on transient transfection methods and lack of suitable stable cell line	Development of stable packaging cell line (e.g., WinPac by Sanber et al. [7] in [270])
2	Use of adherent cell in LV production	Development of suspension cultures McCarron et al. [270] particularly ([98,129,146,170])
3	Low recovery and loss of vector functionality	*The fundamental study and understanding of LV bioprocessing and rational bioprocess development will be crucial to address this.*
4	Lack of international standards for LV vector products	*This is an industry-wide challenge. Product-specifications and process-specifications may vary depending on application.* Review on a case-by-case basis ([270])
5	Difference in purity and potency requirements for in vivo and ex vivo applications	As in No. 4
6	Greater batch-to-batch variability (e.g., ratio of total vs functional vector)	Ensure measurement of both total and functional vectors as well as assessments following cell transduction (e.g., proviral DNA titres, RNA, etc.) ([270]).
7	Inter-assay variation of LV titering between laboratories and operators	*This is a challenge which needs to be addressed at company-level. Implementing automated, analytical assays and comparing to international standards such as WHO LV standard [255]. Training in established protocols and maintaining personnel expertise.*
8	Challenges in testing for replication-competent lentivirus	Development of more sensitive and rapid PCR-based techniques (McCarron et al. [270]).

* Summarised from McCarron et al. [270]. ** Text in italics are the current authors comments.

## Data Availability

Not applicable.

## Abbreviations

The following abbreviations are used in this manuscript:

AEX	Anion Exchange Chromatography
ATMP	Advanced Therapy Medicinal Products
CaPi	Calcium Phosphate
CAR-T	Chimeric Antigen Receptor T-Cell
Cocal-G	Cocal Glycoprotein
ddPCR	Digital Droplet Polymerase Chain Reaction
DEAE	Diethylaminoethyl
DLS	Dynamic Light Scattering
DMEM	Dulbecco’s Modified Eagle Medium
Elisa	Enzyme Linked Immunosorbent assay
GFP	Green Fluorescent Protein
HEK	Human embryonic Kidney
HIV	Human Immunodeficiency Virus
HPLC	High Performance Liquid Chromatography
IEX	Ion Exchange Chromatography
kb	Kilobase
LDL-R	Low-Density Lipoprotein Receptor
LMH	Litres/metres${}^{2}$/hour
LNGFR	Low Affinity Nerve Growth Factor Receptor
LTR	Long Terminal Repeats
LV	Lentiviral Vector
mAb	Monoclonal Antibody
MLV	Murine leukemia virus
MOI	Multiplicity of Infection
MWCO	Molecular Weight Cut Off
p/v	Power /Volume
PBS	Phosphate Buffered Saline
PEG	Polyethylene glycol
PEI	Polyethylenimine
PERT	Product Enhanced Reverse Transcription
PES	Polyethersulphone
PVDF	Polyvinylidene Fluoride
QA	Quaternary Amine
QA/QC	Quality assurance/Quality Control
qPCR	Quantitative Polymerase Chain Reaction
SEC	Size Exclusion Chromatography
SF	Sterile Filtration
TFF	Tangential Flow Filtration
TMP	Transmembrane Pressure
TU	Transducing Unit
UC	Ultracentrifuge
UF/DF	Ultrafiltration/Diafiltration
USD	Ultra Scale Down
VSV-G	Vesicular Stomatitis Virus Glycoprotein
WHO	World Health Organization
